# Interactions of Sea Anemone Toxins with Insect Sodium Channel—Insights from Electrophysiology and Molecular Docking Studies

**DOI:** 10.3390/molecules26051302

**Published:** 2021-02-28

**Authors:** Beata Niklas, Milena Jankowska, Dalia Gordon, László Béress, Maria Stankiewicz, Wieslaw Nowak

**Affiliations:** 1 Institute of Physics, Faculty of Physics, Astronomy and Informatics, Nicolaus Copernicus University, Grudziadzka 5, 87-100 Torun, Poland; 2 Faculty of Biological and Veterinary Sciences, Nicolaus Copernicus University, Lwowska 1, 87-100 Torun, Poland; mjank@umk.pl (M.J.); stankiew@umk.pl (M.S.); 3 Department of Biomolecular Sciences, Weizmann Institute of Science, Rehovot 76100, Israel; dalia.gordon@gmail.com; 4 Department of Internal Medicine, Clinic of Immunology, Division of Experimental and Clinical Peptide Research, Hannover Medical School, 30625 Hannover, Germany; dr.beress@t-online.de

**Keywords:** anemone toxins, sodium channels, fast inactivation, docking, electrophysiology

## Abstract

Animal venoms are considered as a promising source of new drugs. Sea anemones release polypeptides that affect electrical activity of neurons of their prey. Voltage dependent sodium (Nav) channels are the common targets of Av1, Av2, and Av3 toxins from *Anemonia viridis* and CgNa from *Condylactis gigantea*. The toxins bind to the extracellular side of a channel and slow its fast inactivation, but molecular details of the binding modes are not known. Electrophysiological measurements on *Periplaneta americana* neuronal preparation revealed differences in potency of these toxins to increase nerve activity. Av1 and CgNa exhibit the strongest effects, while Av2 the weakest effect. Extensive molecular docking using a modern SMINA computer method revealed only partial overlap among the sets of toxins’ and channel’s amino acid residues responsible for the selectivity and binding modes. Docking positions support earlier supposition that the higher neuronal activity observed in electrophysiology should be attributed to hampering the fast inactivation gate by interactions of an anemone toxin with the voltage driven S4 helix from domain IV of cockroach Nav channel (NavPaS). Our modelling provides new data linking activity of toxins with their mode of binding in site 3 of NavPaS channel.

## 1. Introduction

Voltage dependent sodium (Nav) channels are cell transmembrane proteins responsible for the depolarizing phase of action potentials which are carriers of information in excitable tissues. Nav channel structure consist of a single polypeptide chain that folds into four domains (DI–DIV) with six transmembrane helices (S1–S6) each. In each domain, helices S1–S4 constitute the so-called voltage-sensing-domain (VSD) with helix S4 acting as a voltage sensor. Helices S5 and S6 contribute to the ion conducting pore. Upon membrane depolarization, the outward movement of positively charged S4 helices generates the gating current which triggers the activation of the sodium channel [1]. A particular function is linked with the upward motion of the S4 segment in DIV since it is coupled to the inactivation gate (IG). When S4 helices are raised outwards, the intracellular IG quickly blocks sodium ions entry into the neuron in a process named fast inactivation (completed within 1–2 ms). Due to this blocking mechanism, neurons exhibit very short action potentials and, therefore, enable a high frequency of signal transmission [2]. It is accepted that, in vertebrates, the IG gate, located in DIII-DIV linker, consists of Isoleucine-Phenylalanine-Methionine (IFM) motif [3], but consensus identification of IG in insects, as well as molecular details of Nav channel blocking mechanism by the gate, is still elusive.

**Figure 1:**
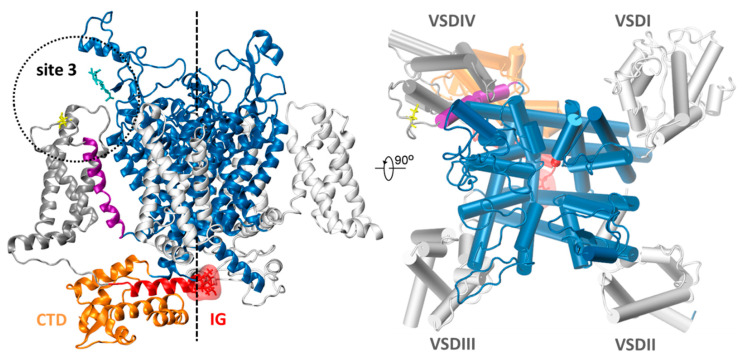
A schematic view of the *Periplaneta americana* voltage dependent sodium channel (NavPaS) based on the Protein Data Bank structure 6A95 [15]. Helices S5 and S6 contributing to the pore formation are shown in blue. Helices S1-S4 forming voltage sensing domains (VSD) are in silver. An approximate location of the pore is indicated by the dashed line. Helix S4 of domain VSDIV is shown in purple. The C-terminal domain (CTD) is presented in orange and located close to it the hypothetical inactivation gate (IG) marked in red (a surface representation) as a part of DIII-DIV linker (red). Toxin binding site 3 region (dotted circle) is delineated by Glu1255 (yellow) and N-acetylglucosamine (NAG) molecule (cyan). In the top view, a single sodium ion is indicated as a red dot.

Many modifications in Nav function result in the disruption of nervous and muscle function and can lead to convulsions, contractive or flaccid paralysis, and even death. In humans, mutations in sodium channels cause several diseases, such as miotonias, myasthenias, epilepsy, and pain and movement disorders [4,5]. Nav channels are quite an old “invention” of evolution [6] and, as such, have become a target for many natural toxins. Years of research on the interaction between sodium channels and natural toxins allowed to classify them into two groups: (1) sodium channel blockers and (2) Nav channel gating modifiers. Toxins bind to seven receptor sites (site 1–site 7) localized in different parts of the Nav protein [7]. Depending on the type of receptor site, toxins induce various modification of the Nav channel function. Toxins that bind to receptor site 3 (site 3 toxins), which is located in the extracellular loop connecting segment S3 and S4 in VSDIV of Nav channel (Figure 1), inhibit the fast inactivation of the sodium channel. Such toxins, usually cysteine-rich peptides [8], were found in the venoms of scorpions [9], spiders [10], sea anemones [11], and venomous sea snails—Conus [12]. Many sea anemone venom neurotoxins immobilize pray and serve as defense against predators. They act by binding to site 3 and inhibiting the fast inactivation phase of Nav channels [13]. Sea anemone toxins are, therefore, of great interest in the research on pain [14] and neuronal conductance modulation.

Since site 3 and IG of Nav channel are located on opposite sides of the cell membrane, in order to affect conductance, the presence of a toxin must be communicated to a distant location in the channel, implying allosteric effects in Nav channels. However, the precise mechanism on the molecular level of changes induced by toxin binding leading to the inhibition of Nav channel inactivation is still elusive. Here, we present data that contribute to a better understanding of the first part of this process, i.e., toxins-Nav channel interactions.

Recent determination of the cryo-electron microscopy (cryo-EM) structure (2.6 Å) of Nav channel from American cockroach, *Periplaneta americana*, (NavPaS) allowed us to perform molecular docking of selected toxins to Nav protein and to monitor toxin-channel interactions [15]. Moreover, based on electrophysiological studies, we were also able to measure effects of these peptides on electrical signaling in isolated cockroach nerves. The aim of our research was to compare interactions of different sea anemone toxins, Av1, Av2, Av3 from *Anemonia viridis* venom (formerly ATX I, ATX II, ATX III from *Anemonia sulcata* venom) and CgNa from *Condylactis gigantea* venom with insect NavPaS channel. Such knowledge will contribute to the understanding of the boundaries of receptor site 3 and may be useful in design of new, natural toxins-derived drugs [16].

## 2. Results

A schematic structure of NavPaS studied here, based on cryo-EM measurements from 2018 [15], is presented in Figure 1. 

### 2.1. Electrophysiology

**Figure 2:**
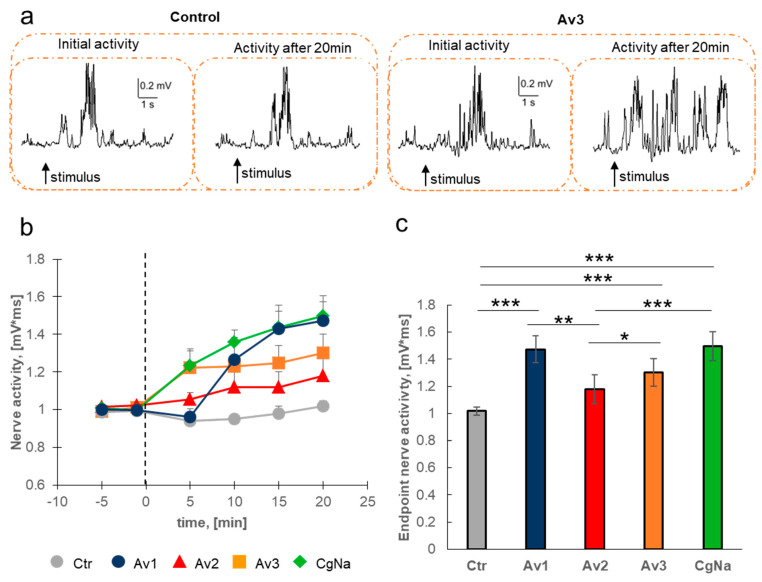
Sea anemone toxins increase the activity of the cockroach (*Periplaneta americana*) cercal nerve. (**a**) Original, representative records of cercal nerve activity are presented for control and after sea anemone toxin (Av3) application. The moment of stimulation is marked by an arrow. (**b**) Normalized nerve activity (measured as a surface under the peaks for response to mechanical stimulation of *cerci*—mV*ms) is presented in time after toxins application. Black dashed line represents the application of toxin in time “0”; the mean of values before toxin application was set as “initial value”. Grey dots represent control values, blue circles represent Av1 toxin, red triangles represent Av2 toxin, orange squares represent Av3 toxin, and green diamonds represent CgNa toxin. (**c**) For clarity, statistical differences between all groups were shown for the endpoint (activity in 20 min after application of toxin). The statistically significant differences between control and tested toxins are marked: * *p* < 0.05, ** *p* < 0.01, *** *p* < 0.001. The data is presented as mean values ± SE, *n* = 8.

The four sea anemone toxins differ in sequence and structure (Supplementary Information (SI), Figure S1); thus, we set to compare their effectiveness in physiological conditions. We used a system for extracellular recordings of isolated cercal nerve activity of the cockroach [17]. Nerve activity was quantified as a size of response to a mechanical stimulus. In control conditions, the level of nerve activity remained constant (94.1–101% of the initial activity) over 20 min of experiment (Figure 2b). 

Sea anemone toxins were expected to increase the cockroach nerve activity because, as it was shown in 1984, they prolong considerably action potentials in isolated giant axons [18]. Indeed, in the cercal nerve preparation, all toxins induced a progressive increase of the response to mechanostimulation (Figure 2). As an example of our experimental results, a comparison of nerve activity between the control and Av3 toxin treated preparations is presented in Figure 2a. Within 20 min after application, Av1 toxin caused an increase of nerve activity to 147.3 ± 9.8% of the initial control value (the Fisher’s least significant difference (LSD) test, d.f. = 138.189, *p* < 0.001, Figure 2b). The structurally similar toxin Av2 caused a much weaker effect. After 20 min, the nerve activity was only 117.9 ± 10.8% of the initial value and did not significantly differ from the control values (Figure 2b). Application of the structurally unique Av3 toxin resulted in an intermediate increase of the nerve activity. The endpoint activity was raised to 130.0 ± 10.1% of the initial value (LSD test, d.f. = 139.035, *p* < 0.001, Figure 2b). The last toxin tested—CgNa—caused the fastest and greatest increase of activity, to 149.6 ± 10.9% of the initial value (LSD test, d.f. = 139.035, *p* < 0.001, Figure 2b). Notably, the effects of Av2, Av3, and CgNa were fast, visible already in the first few minutes of experiments. In contrast, the impact of Av1 was observed with delay, and the first increase of nerve activity was noticeable only after 10 min of toxin application (Figure 2b). 

Among the four toxins tested, the influence of Av2 on the nerve activity was significantly lower than the effects induced by the other toxins (Av1 vs. Av2 d.f. = 138.132, *p* < 0.01; Av3 vs. Av2 d.f. = 139.035, *p* < 0.05; CgNa vs. Av2 d.f. = 139.035, *p* < 0.001; Figure 2c). Thus, the four toxins increased neuronal activity at different efficacies.

### 2.2. Molecular Docking 

A schematic structure of NavPaS used for molecular docking is shown in Figure 1. Anemone toxins were docked using Scoring and Minimization with AutoDock Vina (SMINA) software [19] to the whole sodium channel, but only the lowest energy poses localized in the site 3 region (DIV-DI) (see Figure 1) were further scrutinized. Amino acid sequences of Av1, Av2, Av3, and CgNa may be found in Figure S1 (SI). For docking of Av2 the homology model was used (see Materials and Methods). However, for Av1, Av3, and CgNa, where 8, 28, and 20 NMR alternative structures are published in Protein Data Bank (PDB) (codes: 1ATX, 1ANS, 2H9X), respectively, a selection was necessary. We performed preliminary screening docking in order to determine which alternative NMR toxin structure gives the best SMINA score. For example, in the Av1 set, the best SMINA score was −4.89 kcal/mol, and the worst −4.18 kcal/mol, with a standard deviation in this population of 0.30 kcal/mol. The same data for Av3 NMR structures are −9.72, −7.25, and 0.58 kcal/mol, respectively. These numbers show that a toxin structure flexibility may contribute up to 2.5 kcal/mol in SMINA score, related to the energy of binding. Eventually, for Av1, model 7; for Av3, model 4; for Av3’, (an alternative binding pose of Av3, see below) model 24; and for CgNa, model 19, from PDB database were selected for further analysis. 

**Figure 3:**
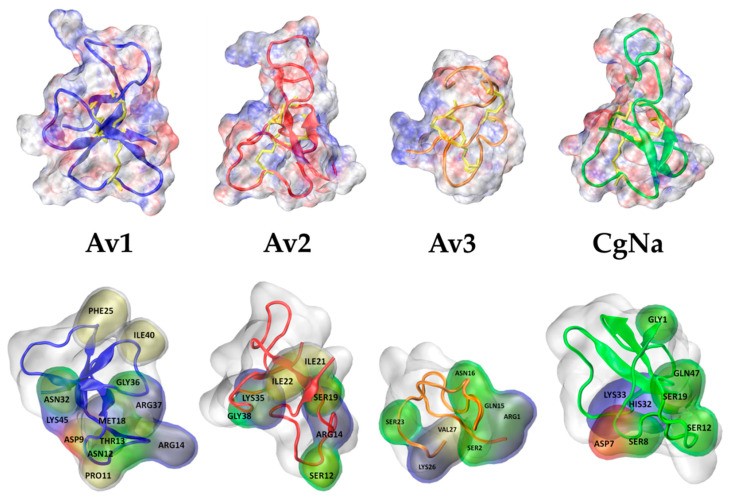
Upper panel: Molecular shapes of anemone toxins studied. In the upper row, electrostatic potential is mapped on a molecular surface; positive = blue, negative = red. In cartoon representation, a peptide backbone is shown, and disulfide bonds are marked in yellow (licorice shapes). Lower panel: Neutral (green), hydrophobic (yellow), and charged (positive = blue, negative = red) residues being in close contacts with NavPaS site 3 residues are indicated.

Structures of the anemone toxins selected are presented in Figure 3. Main residues being in close contacts (distance <2.5 Å) with NavPaS upon docking and their hydrophobic/hydrophilic character are indicated. The charge of the selected residues involved in sodium channel interactions is indicted by coloring in Figure 3. Anemone toxins contain 3–6 charged residues and are, therefore, easily soluble in water. 

**Figure 4:**
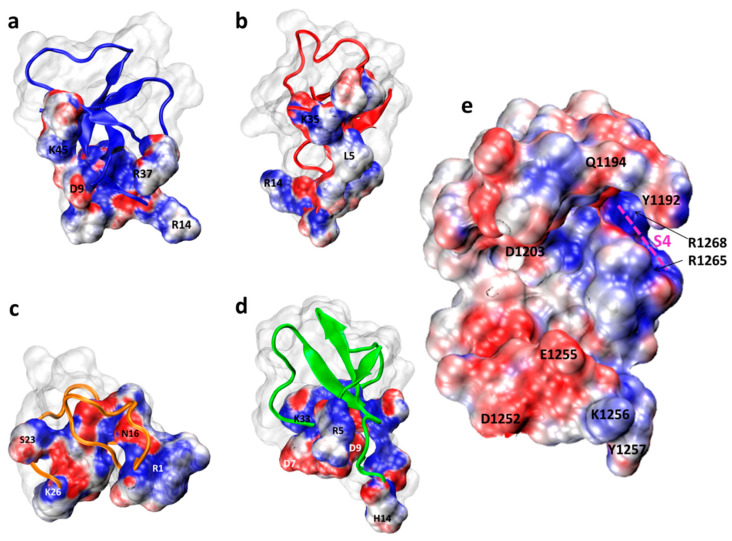
Electrostatic potential maps projected on solvent accessible surfaces of anemone toxins, parts involved in the interface: (**a**) Av1, (**b**) Av2, (**c**) Av3, (**d**) CgNa, and (**e**) the site 3 region of NavPaS visualized from the extracellular side accessible to toxins. In red, negative potential regions are shown, and positive ones are in blue. The neutral regions are white/gray. Note that toxins projections are simply shifted from the docked complexes without mirror reflection. The Visual Molecular Dynamics (VMD) software was used to make these figures [22].

In Figure 4, electrostatic potentials, calculated using the method based on Poisson-Boltzmann equation [20] implemented in PDB2PQR server, Adaptive Poisson-Boltzmann Solver (APBS) module in Visual Molecular Dynamics (VMD) [21], and projected on the solvent accessible surfaces of the interacting toxins’ residues and the site 3 region of NavPaS, are depicted. Coloring facilitates analysis of charges compatibility between toxins (Figure 4a–d) and the site-3 region of NavPaS (Figure 4e).

**Figure 5:**
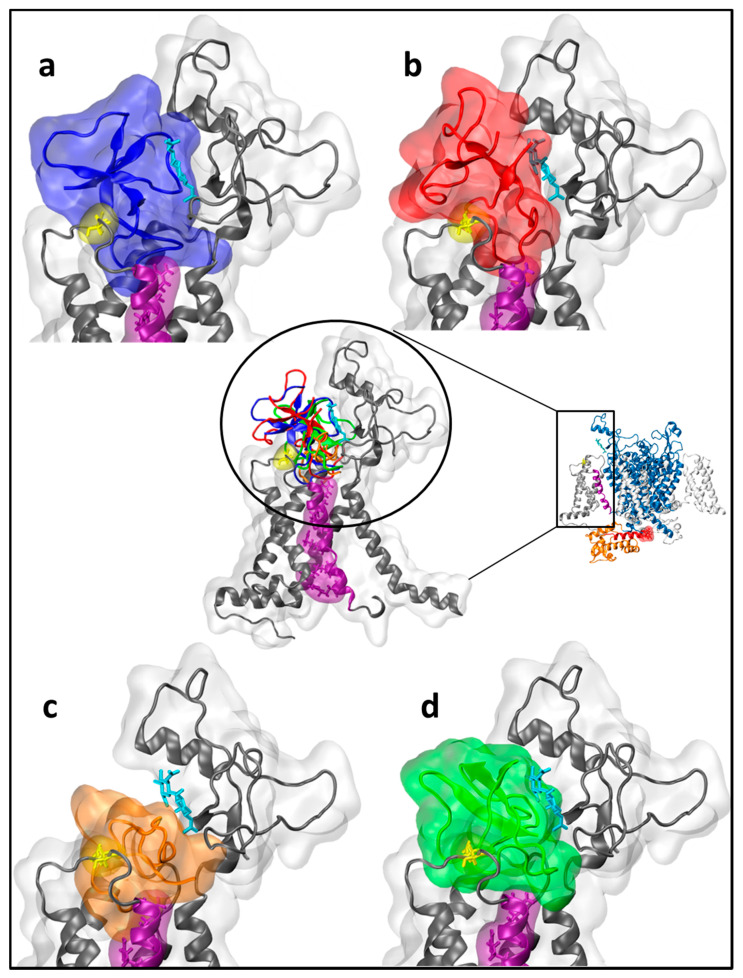
Representations of toxins binding to NavPaS channel site 3: for clarity, only a part of DIV (middle) is presented; (**a**) Av1 = blue, (**b**) Av2 = red, (**c**) Av3 = orange (**d**) CgNa = green. In purple, helix S4 is presented, E1255 is shown in a yellow licorice representation, and N-acetylglucosamine (NAG) is depicted in cyan licorice. A schematic structure of the whole NavPaS structure is presented in the middle right panel.

Toxins are located in a close vicinity of site 3, in the extracellular region of DIV-DI junction (Figure 1, Figure 5). The selected representations showing modes of docking and used for further analysis are presented in Figure 5.

In Table 1, we show parameters calculated to characterize intermolecular interactions of toxins with the channel. SMINA scoring function (SSF) is proportional to the expected binding energy. The negative values indicate that all toxins upon binding are stabilized. Clearly, the binding energy of Av3 is the lowest one (see Table 1, SSF).

Recently introduced Residue-Residue Contact Score function (RRCS) (i,j) is defined as a distance based measure of possible interactions between a particular toxin residue i and a channel residue j [23]. Analysis of RRCS may facilitate studies of individual residues impact on NavPaS function. It is purely structural and static parameter but monitoring RRCSs in different stages of the ion channel “life-cycle” may lead to useful data on allosteric interactions and regulation. Here, RRCS allows for estimation of how tight a toxin is in a contact with the channel and how many residues are potentially involved in direct interactions with the toxin. Moreover, the analysis of RRCS shows how deep into the channel structure toxin effects may extend. The total contact score between toxins and the channel vary from RRCS=86 for Av2 to RRCS=152.8 for Av3 (Table 1). Notably, di-N-glycosylated residue Asn330 from NavPaS is a partner in all toxins binding, but to different degrees (Table 1). We denote this particular glycan side chain as NAG (2-acetamino-2-deoxy-beta-D-glucopyranose). The contact of Av3 with this glycan is significantly lower as compared to the other toxins. RRCS values for all scored residue pairs are available in Table S1 (SI). The non-zero RRCS data presented in Table S1 (in SI) delineate a broad site 3 region. Interestingly, the data presented in Table 1 indicate that all docked toxins are in contacts with S4 segment of DIV. This S4 DIV helix is critically important for the IG control [2]. Upon binding, between 55% (Av2) to 85% (Av3) of all amino acids in toxins are located in the channel-toxin interface region and perhaps participate in the binding. Therefore, the solvent has quite reduced contacts with sodium channel bound toxins. We quantified these arrangements by analyzing Solvent Accessible Surface Area (ASA) values. Parameter ASA_TI (in %) quantifies a percentage of toxin ASA coming from residues which contribute to the toxin-channel interface. In each case, ASA_TI is lower than 50% (Table 1). Notably, the ASA_TI value for Av3 is the highest, perhaps due to its high hydrophobic interacting surface [24] and small size. We also show ASA_NavI (in %)—an analogous parameter—showing a percentage of the whole NavPaS present in the interface. As expected, these are quite small numbers; only 1.2–1.7% of total channel ASA is covered by a toxin after binding. If we take into account only the extracellular part, these percentage will be higher by at least a factor of four. Still, we infer that the binding of toxin only weakly screens the channel from water, and this does not affect channel’s structure. 

We calculated Buried Surface Area (%BSA) for the channel residues Glu1255, Arg1265, Arg1268 which are possibly important for toxins binding and IG dynamics modifications (Table 1). Interestingly, all toxins covered Glu1255, and all are in a contact with top arginines (Arg1265, Arg1268) from voltage sensing helix S4 DIV, supporting their common mode of action on the sodium channel.

The small Av3, in contrast to the other toxins, exhibits four co-localized aromatic residues (Tyr7, Trp8, Trp13, Tyr18) previously suggested to participate in toxin binding [24]. Therefore, we added a relatively low energy pose Av3’ having that region more deeply buried in the channel than the standard, i.e., the lowest energy Av3 structure, for further analysis. Data for the alternative binding pose (Av3’) of Av3 toxin is presented in Table 1. Notably, whereas the RRCS for amino acid residues (AA) is highly similar to Av3 as is the SSF, the RRCS for NAG is significantly lower for Av3’, suggesting weaker interaction with the D1 loop.

It is tempting to search for hot spots in channels structures affecting toxins binding with similar mode of action. A recent study [25] indicated that mutations of a histidine residue in *Drosophila melanogaster* Nav channel, located in analogous position to His392 in NavPaS D1 pore region, affect Av3 toxin modulation of sodium currents. Therefore, we performed several simulations of Av3 toxin docking into the following variants of NavPaS: His392Ala, His392Phe, His392Tyr. Results of docking to those protein variants are presented in Tables S2–S4 and Figure S5. The type of residue in the 392 position of NavPaS affects some close contacts between Av3 and the channel. However, the total RRCS values for Av3-channel residues contacts are similar in wild type (WT) and mutant variants (Table S2). We observed a closer contact of Av3 with NAG of mutated channel, as the pose of the toxin was shifted towards DI domain to which NAG is attached. Positions of Cα of Av3 in the lowest energy pose in docking to H392F (SFF: −9.41 kcal/mol) and H392Y (−9.05 kcal/mol) overlap, with only differences observed in a region of Cys22-Val27. However, this Av3 terminal part gives a high contribution to the contact with S4 of VSDIV. Val27 of Av3 docked to the H392F Nav channel mutant interacts with Arg1268 from S4, which gives BSA value of this Arg equal to 83%. Similarly, high value of BSA for Arg1265 in H392T variant results from contacts with Val27 and Tyr7 of Av3. However, total BSA value for S4 arginines is the highest in the lowest energy pose of Av3 docked to the WT channel. 

Another interesting position in the NavPaS channel is D1252, since mutations in analogous positions in various organisms affected anemone toxin binding [26]. Therefore, we repeated the four toxins docking procedures to D1252E, D1252R, and D1252A channel mutants. SMINA derived binding energies are indeed affected and are collected in Table S5 (SI). The relatively limited changes in toxins interaction to those channel mutants calculated here may suggest that channel mutations might modify its structure under physiological conditions, as have been demonstrated electrophysiologically for Av2 activity on the equivalent to D1252R, monitored on DmNav1-D1701R expressed in oocytes [24].

**Figure 6:**
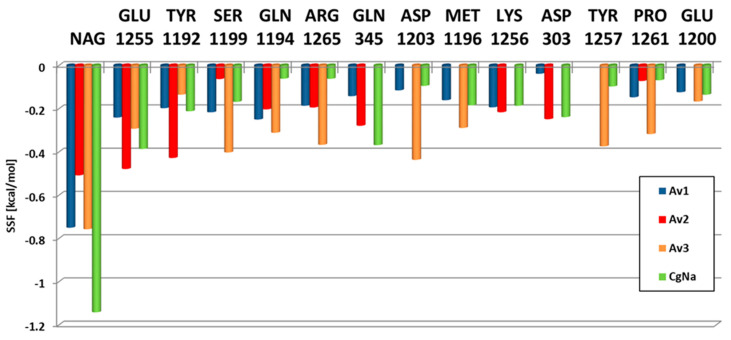
Contributions (in kcal/mol) of NavPaS residues to toxin binding energy measured by the SMINA scoring function (SSF) parameter. The role of each residue varies, depending on the toxin.

We determined what WT channel residues contribute the most to anemone toxin binding energy. Detailed contributions, estimated as values of a decomposed SSF score, are presented in Figure 6. According to our docking results, the NAG side group is a major player in anemone toxin binding. The second most important moiety is Glu1255. On average, Tyr1192, Ser1199, Gln1194, Arg1265 contribute approximately in a similar way to each toxin stabilization. The majority of channel amino acids involved in anemone toxin binding are from DIV domain, only two residues (Gln345, Asp303) are from DI. Notably, the roles of particular channel residues vary between toxins, but the list of channel’s residues involved with substantial contributions to SFF (Figure 6) is limited (13 AA). Interestingly, the smallest toxin Av3 interacts apparently with 7 residues (Ser1199, Gln1194, Arg1265, Asp1203, Met1196, Tyr1257 and Pro1261) more strongly than the other toxins, in accordance with its highest interacting energy SSF (kcal/mol) (Table 1).

Data showing which amino acids from toxins are involved in binding to the NavPaS are presented in Figure S2 (SI). We observed that the toxins in their best poses engage the following residues (contribution to SSF > 1 kcal/mol): **Av1**: Arg14, Thr13, Ile40, Arg37, Pro11, Lys45, Asn12; **Av2**: Ser12, Val13, Asn16, Gln47; **Av3**: Arg1, Val27, Lys26, Trp13, Gln15, Ser23, Tyr18, Asn16, Pro25, Ser2; **CgNa**: Gln47, Trp31, Ser12, Trp23, Val13, Lys33, Gly1, His14, Arg5, His32.

**Figure 7:**
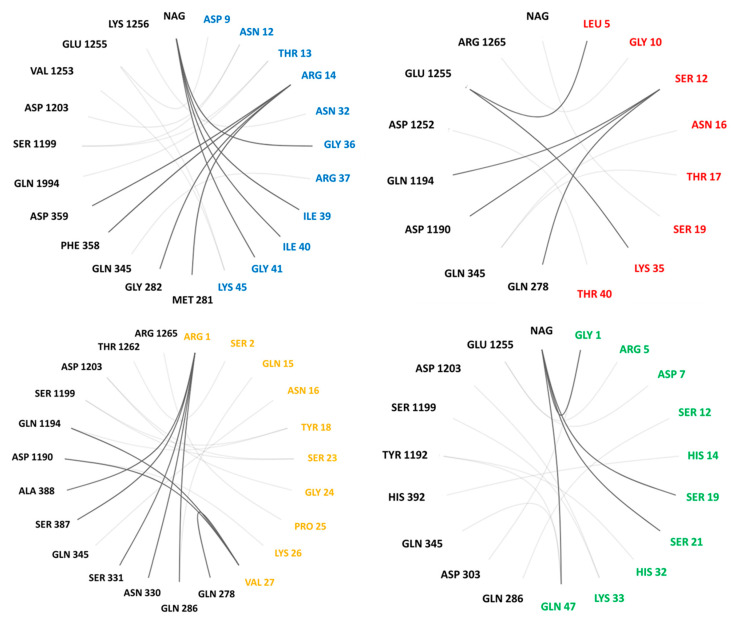
Pairs of residues forming hydrogen bonds and salt bridges between anemone toxins and NavPaS. Av1, Av2, Av3, and CgNa residues are indicated in blue, red, orange, and green, respectively. NavPaS residues are in black. Note that residues from 278–388 belong to DI, the rest to DIV of NavPaS. GetContacts server was used to determine contacts and prepare the plots [22].

Our best docking poses were further analyzed using server GetContacts [22]. Using this server, we determined possible hydrogen bonds and salt bridges between NavPaS and each toxin. Results are presented in Table 2 and Figure 7. This analysis shows that salt bridges are present in all toxins in poses exhibiting the lowest energy: Glu1255-Lys is seen in Av1 and Av2, while Asp-Lys is present in Av3 and CgNa (Table 2). Dominant interactions, shown in Table 2, are hydrogen bonds. Their number vary from 10 (Av2) to 19 (Av3). So, the high number of hydrogen bonds in Av3 (Table 2) correlates with the highest binding energy calculated by SMINA for this toxin (Table 1). Av3 is the smallest peptide, with only 27 amino acids, and because of that, it docks quite deeply in the site 3 region of NavPaS. Notably, in its alternative Av3’ pose two aromatic ring-cation interactions were discovered: Tyr1192-Arg1 and Tyr1204-Lys26. Interestingly, no aromatic residues of Av3 were involved in that type of interactions. Instead, Trp8 and Trp13 from Av3 oriented in the Av3’ pose participate in hydrophobic interactions with some five hydrophobic patches in the channel.

The residues involved in anemone toxin- NavPaS hydrogen-bonded and salt bridges interactions are presented in Figure 7. Interacting pairs in best energy poses were determined by GetContacts server [22]. This presentation highlights the smallest number of Av3-channel interactions, which is in good agreement with our experimental results.

## 3. Discussion

Ion channels are complex multi-domain membrane proteins and, therefore, present a big challenge to structural biology. The first cryo-EM structures of eukaryotic voltage dependent sodium channels were published in 2017 for NavPaS subtype from American cockroach (*Periplaneta americana*) [27] and EeNav1.4 from electric eel (*Electrophorus electricus*) [28]. Those extremely important successes opened up the possibility of (1) defining the structure of other different subtypes of Nav channels, (2) the more detailed studies of the mechanism of Nav channels functioning, (3) study of the effects of point mutations occurring in sodium channels and responsible for many diseases, and (4) determination of the mechanism of interaction between Nav channels and natural and artificial ligands that modify Nav activity. Such structural studies may reveal subtle differences in the Nav channel–ligand interactions for substances with apparently similar mode of action. 

**Figure 8:**
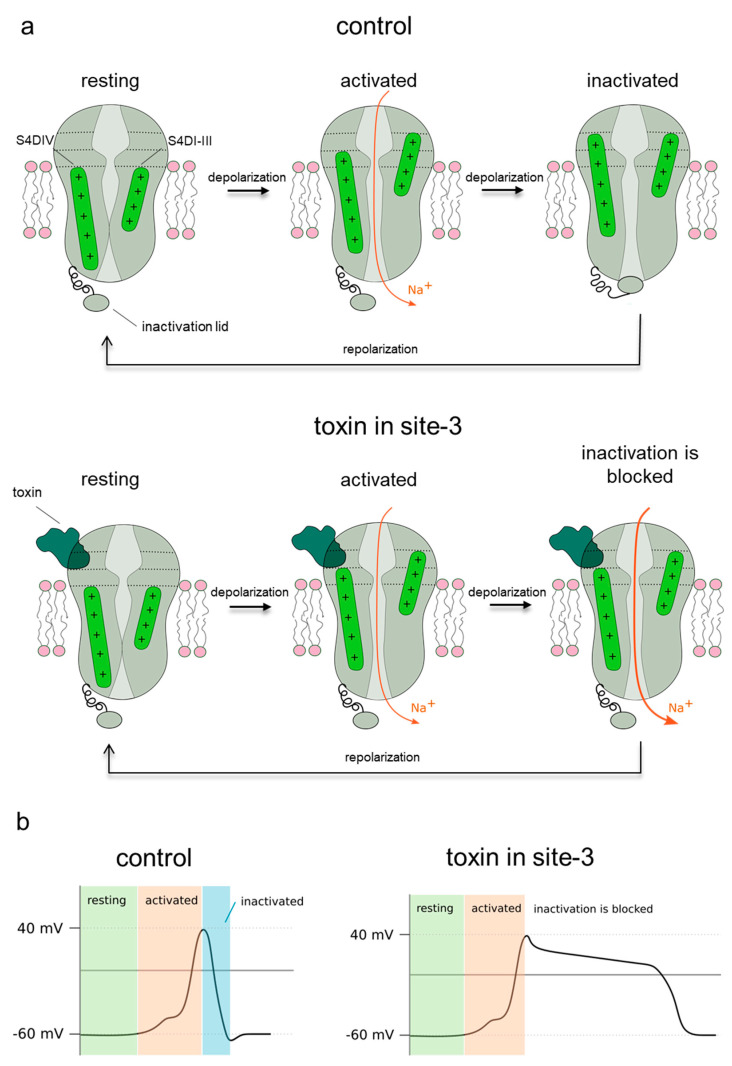
(**a**) Schematic representation of voltage gated sodium channel in control conditions (upper raw) and in the presence of site-3 toxins (lower raw). The inactivation gate and voltage sensors (S4) of domains I-III and domain IV are indicated in green. Depolarization of membrane from resting potential to positive values leads to sodium channel activation, where S4 are moved outward and activation gate is opened, making the sodium channel conductive to sodium ions. Reaching the activation state does not require a full S4 movement in the IV domain. In the next step, S4 of the domain IV makes full outward movement and releases the inactivation gate, which closes the channel. When the membrane potential returns to its resting values (repolarization), all S4s move inward which close channel and push out the inactivation gate from channel pore. In the presence of site-3 toxin (lower panel), the complete movement of S4 in IV domain is not possible, allowing the channel to remain activated, not allowing the inactivation gate to close. As a result, the channels are opened until the membrane potential reaches its resting values. (**b**) Representation of action potential generation in typical, isolated nerve cell: In resting state, the membrane potential is highly negative inside (green area). Depolarization pulse leads to opening of the sodium channels; rapid influx of sodium ion pushes the membrane potential to positive values (red area). Next, fast inactivation blocks the Na^+^ conduction and the membrane potential returns to its resting value due to the outflow of potassium ions (blue area). In the presence of site-3 toxin, the inactivation of channels is inhibited; thus, they remain open and positive membrane potential is maintained (the *plateau* action potential is recorded).

In the present study, we selected four sea anemone toxins [29] to compare their effects on the insect preparation containing voltage dependent sodium channels. All these toxins bind to receptor site 3 region in NavPaS [11] and inhibit its fast inactivation (see Figure 8). However, they differ in several aspects. Av1, Av2, and CgNa are larger peptides (46–47 AA, Type I sea anemone toxin) in comparison to the small Av3 (27 AA, Type III). While Av1 and Av2 are quite homologous [30], the amino acid sequence of Av3 is unrelated to Av1 and Av2 [11] (also see Figure S1 in SI). The 3D structures of these toxins are also different [31]. Type I toxins have a four-stranded, anti-parallel β-sheet linked by three loops with a conserved arginine (Figure 3) and three pairs of S-S bound cysteines [32]. The small Av3 lacks any secondary structure (see Figure 3), having a series of four turns (two type I turns and two γ turns) [33] stabilized into a compact form by three disulfide bridges: Cys3-Cys17, Cys4-Cys11, and Cys6-Cys22. 

All tested toxins induced an increase in the cercal nerve activity recorded under stimulation of the cercal mechanoreceptors (see Figure 2); our results are in accordance with previous research performed on different preparations. Av1, Av2, and Av3 caused repetitive firing in motor axons in the crayfish (*Astacus leptodactylus*) [34]. Av2 induced repetitive activity in the giant axon in situ in the cockroach’s nerve chain (own unpublished data) and in frog skeletal muscle fibers [35]. Another anemone toxin (anthopleurin-B) increased nerve activity in frog spinal cord [36]. 

The excitatory effect of the tested toxins is usually explained by the similar mode of action of toxins that bind to receptor site 3 region on the sodium channel. Av2 and Av3 compete with the site-3 scorpion α-toxin binding to insect neuronal membranes [24,37]. When tested on single cells, they induce a large prolongation of action potential duration (for review, see Reference [11]). Such effect was observed also in an isolated giant axon of a cockroach after application of Av2 (formerly ATX II) and CTX (*Condylactis* toxin) [18]. Prolongation of action potential duration is the consequence of inhibition of sodium channel fast inactivation and an increase in the time constant of Na current decay under depolarization observed with site 3 anemone toxins acting on sodium channels from vertebrates and insects [24,38]. At the single channel level, site 3 anemone toxins prolong the open time of channels and often induce bursting openings [39,40]. It is worth noting that some isoforms of mammalian Nav channel can show quite different sensitivity to sea anemone toxins [41]. Some of them (cloned rNav1.2β_1_, rNav1.4β_1_, rNav1.7β_1_, rNav1.8β_1_ channels) appeared to be completely insensitive to CgNa [32].

In our study, we found differences in the efficiency of toxins to increase the cercal nerve activity (Figure 2). Differences in effective toxin concentration have been previously observed when comparing the effects of Av1, Av2, and Av3 sea anemone toxins on various crayfish neuronal preparations [34,42]. Later, three similar anemone toxins (ATX II, AFT II, Bc-III) were tested on six isoforms of mammalian sodium channels [43]. ATX II differs from AFT II by only one amino acid, and toxin Bc-III has 70% similarity with ATX II. Unexpectedly significant differences were found in dose-response modification of sodium current induced by these toxins [43]. Thus, our studies further support earlier observations that, despite similarity of amino acid sequences and structures, and/or similarity in mode of action, the binding modes of toxins to the sodium channel may vary greatly. In the present study, we tried to clarify this challenging matter.

Molecular modeling (MM) is currently a well-established tool for studying modes of ligand-protein binding, which we recently used for elucidating mosquito repellents–G protein-coupled recetor interactions [44,45]. Similar peptide toxins interactions with various Nav channels were recently assessed using MM [46]. Permeation of ions through a channel is important topic [47,48,49,50] and studied here NavPaS channel was recently analyzed by molecular dynamics (MD) simulations with respect to that, as well [51]. However, to the best of our knowledge, there were no data on Type I nor Type III anemone toxin docking to insect sodium channels.

Our SMINA based protocol (see Materials and Methods) enabled us to detect that all anemone toxins had low energy poses in a relatively limited fragment of NavPaS exposed to the extracellular medium (Figure 5). The common feature is “capping” S4 VSDIV helix by the docked peptides. Molecular electrostatic potential maps presented in Figure 4 show that binding is rather dominated by electrostatic forces and hydrophobic interactions have a minor role. All toxins form salt bridges between their positively charged lysine residues and negatively charged channel amino acids (Table 2). However, these critical interactions are not identical; Av1: Lys45-Glu1255, Av2: Lys35-Glu1255, Av3: Lys26-Asp1190, CgNa: Lys33-Asp1203. This is in a good agreement with experimental observations underscoring the role of lysines from C-terminal toxin region [13]. Data in Table 1 and Table 2 shows that Av1, Av2, and CgNa are quite well stabilized by interactions with the channel residues. The value of SFF, being on the order of -5 kcal/mol, cannot be directly converted into a binding constant since the solvent effects were neglected in our docking; however, it shows similar propensity of these three toxins to the site 3 region. 

In Av1, in accordance with previous observations [52], we observe strong involvement of Arg14 hydrogen bond and salt bridge interactions with domain DI region (Met281, Gly281; Phe358, Asp259; Table 2). Interestingly, Arg37 of Av1, involved in interactions with DI through Gln345 (Table 2 and Figure S2), is unique for Av1 sequence in comparison with other type I anemone toxins (see SI in [31]).

Av2 exhibited the lowest ability in modulation of neuronal activity in the cockroach neuronal preparation (Figure 2, Table 1). Its binding to the channel seems to be the weakest one, despite similar to other toxins SSF value, since RRCS of 86 is clearly much below the next smallest calculated value of 119 (CgNa). The surface of contact with the channel (ASA_TI) is only 22%, while, in the other toxins, it is in 34–46% range (Table 1). In Av2 the smallest number of hydrogen bonds is observed (Table 2). Interestingly, Ser12 seems to be a major player here, interacting both with DI (Glu28) and DIV (Asp 1190, Glu1194) residues. This residue was not denoted previously as important for Av2 binding in mutagenesis study [38].

CgNa has 47 amino acids and also has interactions (via Arg5, Table 2) with Glu1255 of NavPaS, which seems as most important for anemone toxins interaction. In this toxin interactions with NAG (via Gly1, Ser19, Thr21, Gln47) are particularly strong. A special role of Lys33 bound both to Ser1199 and Asp1203 is also visible. Despite a relatively small direct contact of CgNa with S4 (Table 2), this toxin is quite well stabilized. In the predicted position, it may block helix’s S4 further motion “up” and prevent the fast inactivation of NavPaS.

A much better energetic stabilization of Av3 toxin (SFF ~ −9 kcal/mole) stems from the deeper localization of this small peptide in the DIV cleft. The number of NavPaS residues being in contact with Av3 is the highest in the series, and the total RRCS score (152 vs. 122 and 119 for Av1 and CgNa, respectively) is the highest, as well (see Figure 7, Table 2, Table S1 in SI). Parameters, such as a number of hydrogen bonds, RRCS, and BSA, obtained for Av3 (Table 1) indicate that Av3 exhibits the strongest interaction with S4. Due to its relatively small size, together with loose contacts with Glu1255 and NAG, it is tempting to speculate that Av3 might be partially moving together with S4 upon increase/decrease of the membrane potential. This may explain why the effect of Av3 on insect preparation electrical activity was weaker than that of Av1 and CgNa (Figure 2).

Av3 toxin affects specifically arthropods, while mammalian brain Nav1.2a channels are insensitive to this toxin. In a recent paper [25] mutagenesis studies suggested that in the *Drosophila* DmNav1 channel Trp404 and His405 localized near the membrane surface in D1 are a part of the channel receptor site interacting with Av3. The sequence of insect channels in this region is highly conserved (see Figure S4 in SI) and differs from mammalian Nav channels. Using our protocol, we docked Av3 to His392Ala, His392Phe, His392Tyr variants of NavPaS. The SSF score is similar to those obtained in docking to WT channel (Table S2 in SI) and poses are in the site 3 region, as well (Figure S5 in SI). Analysis of Av3 binding modes (Figure S5 in SI) and toxin-channel close contacts (Tables S3 and S4 in SI) indicate that Av3 indeed has low energy (−9 kcal/mol) poses, being in contact with that DI/SS2–S6 linker region. However, the differences in the calculated Av3 binding energies alone do not explain diverse effects of the toxin on those mutants in a chimeric Nav channel [25], suggesting that perhaps dynamic toxin-channel interactions are missed in our docking models.

By combining results of computational analysis, we selected the most important residues that contribute to anemone toxins binding to NavPaS. For Av1 these are: Asp9, Asn12, Thr13, Arg14, Arg37, and Lys45. For Av2, we found **Leu5**, Gly10, Ser12, **Asn16**, Thr17, Lys35, and Thr40 to have the substantial role. This is in a partial agreement with mutagenesis studies results [38], in which Val2, **Leu5**, **Asn16**, Leu18, and Ile41 were indicated as key players in the binding. Notably, our Thr17 and Thr40 are in the same region of Av2 as Leu18 and Ile41, noted in experiments as important. The involvement of Arg14 in Av2 binding, discussed in [38], is observed only in one of higher energy poses of this toxin. It is plausible that dynamic changes of the toxin in solution upon binding may differ from the toxin structure in our MM conditions, which may account for some of the differences observed. Analysis of CgNa docking (Table 2, Figure S2) showed that Gly1, Arg5, Asp7, Ser12, His14, Lys33, and Gln47 are the most important CgNa amino acid residues in NavPaS binding. Majority of them are polar or charged residues.

A more complex analysis was required for Av3 toxin, as we found two low energy poses (using different NMR models) that are favorable in terms of electrostatic potential (Av3, model 4) or hydrophobicity (Av3’, model 24). Moran et al. [24] showed that mutation of **Arg1**, **Tyr7**, **Trp8**, Pro12, **Trp13**, **Tyr 18**, Glu20, and Ser23 decreased toxin binding affinity to cockroach neuronal membranes. In our docking study, we found five of these residues (shown in bold) as crucial in binding to NavPaS, distributed in Av3 and Av3’ poses. For Av3, we selected **Arg1**, Ser2, Gln15, Asn16, **Tyr18** and Val27 and for Av3’ **Arg1**, **Tyr7**, **Trp8**, Gly9, **Trp13**, Gly24, Lys26, and Val27. Indeed, our pose Av3’ have aromatic residues buried deeply in the channel groves. We suggest that, in native NavPaS channels, both binding poses (Av3, Av3’) are possible, and both may modulate electrical activity, but in Av3 pose the toxin may have stronger effect on IG through stronger interactions with S4 (see Table 1).

*Possible Hot Spots in Toxin Binding to Site 3 of Sodium Channels*

In previous experimental research on sodium channels residues critical for toxin binding, the role of a negatively charged aspartic acid residue, the equivalent of NavPaS Asp1252, have been examined. This residue, located in S3–S4 loop of VSDIV, is conserved in insects and some mammals. Mutagenesis study performed on *Xenopus* oocytes expressing *Drosophila melanogaster* Nav (DmNav1) channel showed that substitution of aspartic acid to arginine (D1701R) abolished the effects of Av2 and scorpion site-3 toxin LqhαIT [24]. In contrast, the DmNav1^D1701R^ mutation has only minor effect on Av3 activity. In the rat brain rNav1.2a channel, which is insensitive to Av3 and LqhαIT toxins, there is glutamic acid in the equivalent position. A single substitution E1613D was found to convert rNav1.2a channel from being insensitive to highly sensitive toward scorpion LqhαIT toxin [53]. Interestingly, a single substitution of aspartic acid to glutamic acid in the equivalent position in rat skeletal muscle rNav1.4 channel (D1428E) decreased the effect of LqhαIT [54]. In our molecular docking study, we observe that Asp1252 slightly contributes to the binding energy (SSF) of Av2 (−0.75 kcal/mol) and Av1 (−0.1 kcal/mol) but does not participate in Av3 and CgNa binding. Asp1252 can also form a hydrogen bond with Thr40 of Av2. The higher affinity of Av2 than that of Av3 toxin to NavPaS Asp1252 is in good agreement with mutagenesis studies [24]. 

The effect of channel mutation D1252X varied between investigated toxins (Table S5 in SI). We observed the most striking impact in Av1, binding of which to the site 3 region of NavPaS mutants was completely abolished (see Table S5 in SI). The D1252R mutation in channel increased the SSF energy of Av2 binding with respect to WT by 0.32 kcal/mol. Although this change in SSF value is limited, dissociation rate of toxin could be increased due to steric interactions in response to depolarization followed by S4 movement [26]. Surprisingly, our D1252R mutation of NavPaS improved CgNa binding by 0.3 kcal/mol, while no effect was observed in binding to neither D1252E nor D1252A mutant variants. Results of SMINA values for D1252X variants should be interpreted with caution, since here we take into account only local, limited to D1252 site, relaxation of NavPaS structure. Possible large scale structural effects of mutations are not included in the modeling. There is also no experimental data providing D1252X structures. 

However, our docking results clearly suggest that the most important amino acid residue for the investigated toxins binding is Glu1255 (Figure 6, Table 2). Notably, this residue is conserved in insect and some mammalian Nav channels. Substitution of corresponding Glu by Gln (E1589Q) in human Nav1.7 channel reduced the effects of CvIV4 scorpion toxin [55] and selectively decreased ProTx-II ability to induce sustained currents around 6-fold [56]. Experimental studies on mammalian Nav1.2a channels revealed that substitution of corresponding Glu by Gln or Arg (E1616Q, E1616R) both significantly decreased affinity for Av2 but not for scorpion α-toxin LqTx [26]. Although sea anemone toxins and scorpion α-toxin bind to overlapping sites, non-identical amino acids of site 3 are crucial for their activity [57].

*Possible NavPaS Residues Affecting Anemone Toxin Binding*

We found that receptor site 3 on the NavPaS channel comprises a broad region in domains DIV and DI, based on the combined data collected in Table 2, Tables S1a and S3 (SI), and Figure 7. The most detailed information come from RRSC which shows close contacts between toxin and channel atoms without assessing any particular physical interaction. The contacts result from calculated optimum docking poses. The broad site 3 region of cockroach NavPaS channel found in this study, based on RRCS cutoff of 1.0, encompasses the following residues:

- DI: [**S5:** Gln278, Ile279], [**EC3:** Met281, Gly282, Val283, Gln286, Phe301, Asp303, Trp306, Phe307, Gly329, Asn330, NAG1601, NAG1602, Ser331, Gln345, Tyr347, Phe358, Ap359], [**EC4:** Ser387, Ala388, His392]
- DIV: [**S1–S2 loop**: Asp1190, His1191, Tyr1192, Gly1193, Gln1194, Met1196, Ser1199, Glu1200], [**S2:** Leu1202, Asp1203, Tyr1204, Asn1206], [**S3:** Gly1247, Leu1248], [**S3–S4 loop:** Asp1252, Val1253, Ile1254, Glu1255, Lys1256, Tyr1257, Phe1258, Ile1259, Pro 1261, Thr1262], [**S4:** Leu1264, Arg1265, Arg1268].

Important observation from the present analysis is the role of NAG group in anemone toxin binding. NAG1601-NAG1602 form a part of site 3 region (Figure 5). Glycosylation in a position corresponding to Asn330 of NavPaS is conserved in human Nav channels and was recently found to play a role in scorpion AaH2 toxin binding [58]. Increased potency of AaH2 for Nav1.2 over Nav1.7 was linked to the fact that Nav1.2 have glycosylated Asn residue in site 3, while Nav1.7 do not have such modification [58]. It is known that glycosylation is a tool of evolution [59] and affects sodium channels function [60]. It would be interesting to know whether the presence of NAG in places corresponding to the site 3 of NavPaS has any special biological role. Experimental studies focused on NAG might resolve this issue. 

The mechanism of anemone toxin impact on NavPaS channel inactivation discussed earlier for site 3 toxins [2] is summarized in Figure 8, and refers to our data, as well. 

Now, we ask the basic question: should we expect the full agreement between our modeling and mutagenesis studies [24,38], since both NavPaS channel and toxins’ structures are known? The answer is not so straightforward. If one assumes, that the anemone toxins “attack” sodium channels in a single and unique mode, then our results seem to be disappointing. However, toxin peptides are partially flexible structures. NMR results in up to 24 alternative structures [33], with RMS distance up to 2 Å. Our SMINA rigid docking shows that binding energies of Av3 and Av3’ differ by less than 1 kcal/mol. Thus, anemone toxins are able to dock in heterogeneous way, and exact distributions of the poses may depend on experimental conditions.

Our MM results are based on several assumptions. One should remember that the SMINA scoring functions, being universally accepted, is one of many theoretical models for toxin-channel binding. For example, other approaches may give different distributions of electrostatic potentials (Figure 4). Since the peptides are quite rich in hydrophilic residues (see Figure 3) we have assumed that water contributes to all toxins in the same way. However, solvent effects may preferably stabilize some toxin poses. The static NavPaS channel structure adopted here is also approximate. We assume that the structure (PDB 6A95) used here, resembles the most abundant natural resting state of this insect channel, but during the working cycle of NavPaS distinct conformations of the site 3 region might be envisaged. Data of RRSC presented in Table S1 should be helpful in tracing allosteric effects in inactivation of sodium channels [58]. How the presence of toxin in the extracellular part of the channel mechanically affects IG located about 80 Å apart cannot be deduced from our docking studies, yet. So, performing extensive molecular dynamics simulations may bring new data on this fascinating but complex systems. We plan to perform such investigations in our lab. 

## 4. Materials and Methods 

### 4.1. Electrophysiology

*Material:* Electrophysiological experiments were performed on adult, male American cockroaches (*Periplaneta americana*). Insects were reared in our own colony, kept at 29 ± 2 °C, fed with oat flakes, apples, and dog food and water ad libitum. Twenty-four hours before the experiment, the cockroaches were moved to room temperature (21 ± 1 °C). 

*Chemicals:* Physiological saline was prepared with 210 mM NaCl, 3.1 mM KCl, 5 mM CaCl_2_, 5.4 mM MgCl_2_, 5 mM Hepes. pH was adjusted to 7.4 with NaOH. All chemicals were purchased from POCH. SA., Gliwice, Poland. The toxins Av1 and Av2 and Av3 were isolated from sea anemone *Anemonia viridis* venom (formely ATX I, ATX II, ATX III from *Anemonia sulcata*) [61], CgNa toxin was isolated from the *Condylactis gigantea* venom [62]. Lyophilized toxins were dissolved in physiological saline to 0.1 mM concentration and then diluted to 1 µM. 

*Electrophysiological experiments*: To determine the effects of anemone toxins on the bioelectrical activity of the cercal nerve, the extracellular recordings were conducted as previously described [17]. Abdominal part of cockroach’s escape system was isolated from insect body. The preparation consisted of two *cerci*, cercal nerves, terminal abdominal ganglion, and short part of connective nerves. In the experimental chamber (3.5 cm Petri dish), the preparation was slowly perfused with physiological saline, while *cerci* were kept dry. Compound bioelectrical activity of cercal nerve was recorded using extracellular electrodes (Alpha Omega Engineering LTD, Nof HaGalil, Israel). Signals were amplified by a differential amplifier, observed at oscilloscope (Hameg 507, Hameg Instruments Gmbh, Mainhausen, Germany) and stored in a computer. Data were analyzed using modified Hameg software. 

During each recording at first the spontaneous (“resting”) activity was recorded during 40 ms. Then, mechanoreceptors covering the *cercus* were stimulated with gentle air puffs, generated by loudspeaker membrane movements controlled by impulse generator with 0.4 Hz frequency. The response to *cercus* stimulation was seen as an increase of cercal nerve activity, appearing just after the stimulation. Usually the response was well defined and its size was estimated as the area under the response peaks. Nerve activity returned to a resting level up to 50 ms (Figure 2a). Each preparation was allowed to stabilize for 10 min before the activity registration. The initial activity was recorded for 5 min and then the physiological saline (in control) or toxin was applied at a concentration of 1 µM. The effects of the toxin were recorded for 20 min.

The treatment effect with anemone toxins on nerve activity was tested by a one-way generalized linear mixed model (GLMM). We included measurement time (minutes of the test) as a continuous variable and replicate as a random factor. Nerve activity was used as a dependent variable while toxin (Av1, Av2, Av3, CgNa) as main factor. Each analysis was followed by multiple comparisons using Fisher’s LSD post hoc test. Analysis was conducted in the IBM SPSS 25 Statistics software (IBM Corporation, Armonk, NY, USA). The results were expressed as mean values ± SE. The differences were considered as significant when *p* < 0.05.

### 4.2. Molecular Modeling and Docking

We performed molecular docking of four toxins to the *Periplaneta americana* voltage-gated sodium channel (PDB: 6A95) using SMINA package [19], a fork of Autodock Vina [63] that provides enhanced support for minimization and scoring. After removing of spider toxin Dc1a present in the original structure. we carried out a single rigid docking run for each NMR derived model of CgNa (PDB: 2H9X), Av1 (PDB: 1ATX), and Av3 (PDB: 1ANS) and selected two best scored models for further studies of each toxin. Then, we performed next round of 5 independent rigid docking runs per each model using default SMINA settings, generating up to 100 poses per run. We build a homology model of Av2 toxin using SWISS-MODEL structure homology-modeling server [64] with the following structures of sea anemone toxins as templates based on highest sequence homology structures with PDB codes: 1AHL, 1APF, 1SHI. For the best scored homology model of Av2, we performed 10 independent docking runs. Thus, we have obtained more than 5000 docking poses. We added hydrogen atoms using CHARMM-GUI server [65] and then analyzed and visualized results with the VMD code [21] and home-made scripts.

A residue-residue contact score (RRCS) is an atomic distance-based parameter that quantifies the strength of contact between residue pairs by summing up distances in all possible inter-residue heavy atom pairs [23]. For each docked toxin, we calculated RRCS values using the python script provided by Zhou et al. and further analyzed data with NumPy package. We calculated the Accessible Surface Area (ASA), Buried Surface Area (BSA), and percentages of residues corresponding to the toxin-channel interface using PDBePISA server [66]. GetContacts [22] server was used to identify toxin-channel interaction residues and a character of those interactions (hydrogen bonds, salt bridges, etc.). To create maps of electrostatic potential for toxins, we used PDB2PQR server [67] and APBS in VMD [21].

## 5. Conclusions

Electrophysiological experiments revealed different effects of four sea anemone toxins on *Periplaneta americana* neuronal preparations activity. Av1 and CgNa are the most potent toxins affecting inactivation process of sodium channel, while Av2 has the lowest impact on inactivation.

Our molecular docking with SMINA software [19] provides firm arguments that Av1, Av2, Av3, and CgNa bind in site 3 extracellular part of NavPaS channel. The low energy modes of binding prefer surfaces of toxins that fit the best in terms of a number of hydrogen bonds and salt bridges to the channel surface. We noticed that hydrophobic contacts play less significant role in sea anemone binding to NavPaS. We observe moderate compatibility of electrostatic potentials surfaces between all four toxins and the site 3 NavPaS region. The contact areas toxin-channel moderately correlate with activity modulation effect observed in electrophysiology measurements in cockroach neurons. The docking poses obtained support the molecular model in which the upward motion of S4 helix in DIV domain is hampered by the presence of the anemone toxin in site 3 (Figure 8). The inactivation gate, in this case pivoted by the Alanine-Threonine-Aspartic acid (ATD) triad in the DIII-DIV linker, upon application of any toxin studied here, is locked in an intermediate position and cannot complete the fast inactivation cycle. Docking provided various sets of residues affected by formation of sea anemone toxins and sodium ion channel complex (Table S1 in SI of RRCS). One may expect that mutations in these sites will affect functioning of NavPaS. In several cases, there is a reasonable correspondence between our predicted “hot spots” and earlier mutagenesis based experimental studies [11]. The lack of full agreement is justified by expected heterogeneity in anemone toxin binding modes in physiological conditions. Due to the overall high similarity of human and NavPaS sodium channel structures, the analogous critical regions in the human proteins may be now identified. Such data should facilitate tracking genetic effects in channelopathies.

## Figures and Tables

**Table 1:** Parameters (*) characterizing interactions of anemone toxins with NavPaS channel.

Parameter *	Av1	Av2	Av3	Av3’	CgNa
*Activity in experiment*	$$$	$	$$	$$	$$$
SSF (kcal/mol)	−4.89	−4.98	−9.72	−8.85	−5.53
Total RRCS (AA)	122.10	86.00	152.80	145.90	119.13
Total RRCS for NAG	26.63	19.90	13.90	2.53	38.08
RRCS S4 only	14.80	7.52	21.84	6.51	10.14
Toxin AA in interface (%)	61	55	85	78	70
NAG atoms in interface (%)	68	75	50	46	86
ASA_TI (%)	34	22	46	45	37
ASA_NavI (%)	1.40	1.20	1.40	1.40	1.70
% BSA Glu1255	98	74	43	89	72
% BSA Arg1265 (S4)	77	54	94	31	35
% BSA Arg1268 (S4)	27	0	83	22	0
% BSA NAG	35	42	25	20	42

(*) for description of parameters, see the text.

**Table 2:** Residue (#NavPaS)-residue (#toxin) interactions responsible for anemone toxin binding to NavPaS channel.

Interaction	Av1	Av2	Av3	Av3’	CgNa
Type					
**salt bridge**	ASP 359-ARG 14	GLU 1255-LYS 35	ASP 1190-LYS 26 *		ASP 1203-LYS 33
	GLU 1255-LYS 45 *				
**π-cation**				TYR 1192-ARG 1	HIS 392-HIS 14
				TYR 1204-LYS 26	TYR 1192-HIS 32
**H bond**	sb MET 281-ARG 14	ss GLN 278-SER 12	ss GLN 286-ARG 1	sb ILE 279-TRP 13	ss GLN 286-SER 12
*s—side chain*	sb GLY 282-ARG 14	ss GLN 345-ASN 16	ss GLN 286-GLN 15	sb HIS 1191-ARG 1	sb ASP 303-GLY 1
*b—backbone*	ss GLN 345-ARG 37	ss GLN 345-THR 17	ss ASN 330-ARG 1	sb GLY 1993-ARG 1	ss TYR 1192-GLN 47
	sb PHE 358-ARG 14	ss ASP 1190-SER 12	sb SER 331-ARG 1	sb GLN 1994-ARG 1	ss SER 1199-LYS 33
	sb ASP 359-ARG 14	sb GLN 1194-SER 12	ss SER 331-ARG 1	sb GLU 1200-GLY 24	ss ASP 1203-LYS 33
	ss ASP 359-ARG 14	ss ASP 1252-THR 40	sb GLN 345-ASN 16	sb ASP 1203-LYS 26	sb GLU 1255-ARG 5
	ss GLN 1194-THR 13	sb GLU 1255-LEU 5	sb SER 387-ARG 1	ss ASP 1203-VAL 27	sb GLU 1255-ASP 7
	ss SER 1199-ASN 12	ss GLU 1255-LYS 35	bb ALA 388-ARG 1	sb LEU 1248-TYR 7	b NAG-GLY 1
	sb SER 1199-THR 13	sb ARG 1265-GLY 10	ss ASP 1190-LYS 26	sb GLU 1255-GLY 9	s NAG-HYP 3
	ss ASP 1203-ASN 12	s NAG-SER 19	ss GLN 1194-TYR 18		s NAG-THR 21
*Av3’*	sb VAL 1253-LYS 45		sb GLN 1194-VAL 27	*VAL 283-TRP 13*	s NAG-GLN 47
*hydrophobic*	ss GLU 1255-ASP 9		ss SER 1199-TYR 18	*ALA 388-TRP 13*	
	ss GLU 1255-LYS 45		sb SER 1199-SER 23	*PHE 1258-TRP 8*	
	ss LYS 1256-ASN 32		ss ASP 1203-SER 23	*ILE 1259-TRP 8*	
	b NAG-GLY 36		sb ASP 1203-GLY 24	*PRO 1261-TRP 8*	
	b NAG-ILE 39		sb THR 1262-SER 2		
	b NAG-ILE 40		ss THR 1262-SER 2		
	b NAG-GLY 41		sb ARG 1265-PRO 25		
			s NAG-ASN 16		

## Data Availability

The data presented in this study are openly available in FigShare https://doi.org/10.6084/m9.figshare.14128502.v1, accessed date 27 February 2021.

## Supplementary Materials

The following are available online, Figure S1: Multiple sequence alignment of investigated anemones toxins: Av1, Av2, Av3 and CgNa, Figure S2: Contributions of toxins residues to the binding energy to NavPaS, Figure S3: Binding mode of Av3 alternative pose–Av3’ in the NavPaS site 3 region, Figure S4: Multiple sequence alignment of DI/SS2-S6 region of selected arthropod voltage-gated sodium (Nav) channels, Figure S5: Representations of Av3 toxin binding modes to the site 3 of NavPaS channel H392X mutant variants, Table S1a: Residue-Residue Contact Score (RRCS) values for possible toxin-NavPaS channel (amino acid residues) contacts, Table S1b: Residue-Residue Contact Score (RRCS) values for possible toxin-NavPaS channel NAG contacts, Table S2: Parameters characterizing interactions of Av3 anemone toxin with NavPaS channel wild type (WT) and H392X mutant variants, Table S3: Residue (# H392X NavPaS)-residue (#Av3 toxin) interactions responsible for anemone toxin binding to NavPaS mutant variants channel, Table S4a: Residue-Residue Contact Score values for possible Av3 toxin-NavPaS channel H392X variants (amino acid residues) contacts, Table S4b: Residue-Residue Contact Score values for possible toxin-NavPaS channel NAG contacts, Table S5: Values of SMINA Scoring Function (kcal/mol) for anemones toxins binding to NavPaS channel variants (the lowest energy poses of 5 repetitions up to 100 poses).

## Sample Availability

Docked anemone toxin structures are available from the authors upon a reasonable request.
