# Supplementary material for: Interactions of Sea Anemone Toxins with Insect Sodium Channel—Insights from Electrophysiology and Molecular Docking Studies
Source: Molecules. 2021 Feb 28;26(5):1302. doi: 10.3390/molecules26051302 (PMC7957711; doi:10.3390/molecules26051302)
Supplement: Supplementary file 1 [file molecules-26-01302-s001.pdf]

Supplementary information

Article

Interactions of Sea Anemone Toxins with Insect Sodium Channel—Insights from Electrophysiology and Molecular Docking Studies

Beata Niklas [1,*], Milena Jankowska [2], Dalia Gordon [3], László Béress [4], Maria Stankiewicz [2] and Wieslaw Nowak [1,*]

[1] Institute of Physics, Faculty of Physics, Astronomy and Informatics, Nicolaus Copernicus University, Grudziadzka 5, 87-100 Torun, Poland
[2] Faculty of Biological and Veterinary Sciences, Nicolaus Copernicus University, Lwowska 1, 87-100 Torun, Poland
[3] Weizmann Institute of Science, Rehovot, Herzl 234, Israel
[4] Department of Internal Medicine, Clinic of Immunology, Division of Experimental and Clinical Peptide Research, Hannover Medical School, Hannover, Germany
* Correspondence: beata.niklas@doktorant.umk.pl (B.N.); wiesiek@umk.pl (W.N.)

```
Av1  GAACLCKSDGPNTRGNSMSGTIWVFGCPSGWNNCEGR-AIIGYCCKQ---   46
Av2  GVPCLCDSDGPSVRGNTLSGIIWLAGCPSGWHNCKKHGPTIGWCCKQ---   47
CgNa GVPCRCDSDGPSVHGNTLSGTVWVGSCASGWHKCNDEYNIAYECCKE---   47
Av3  ----------------------RSCCPCYWGGCPWGQNCYPEGCSGPKV   27
                               * . *  *          *.
```

Figure S1. Multiple sequence alignment of investigated anemones toxins: Av1, Av2, Av3 and CgNa.

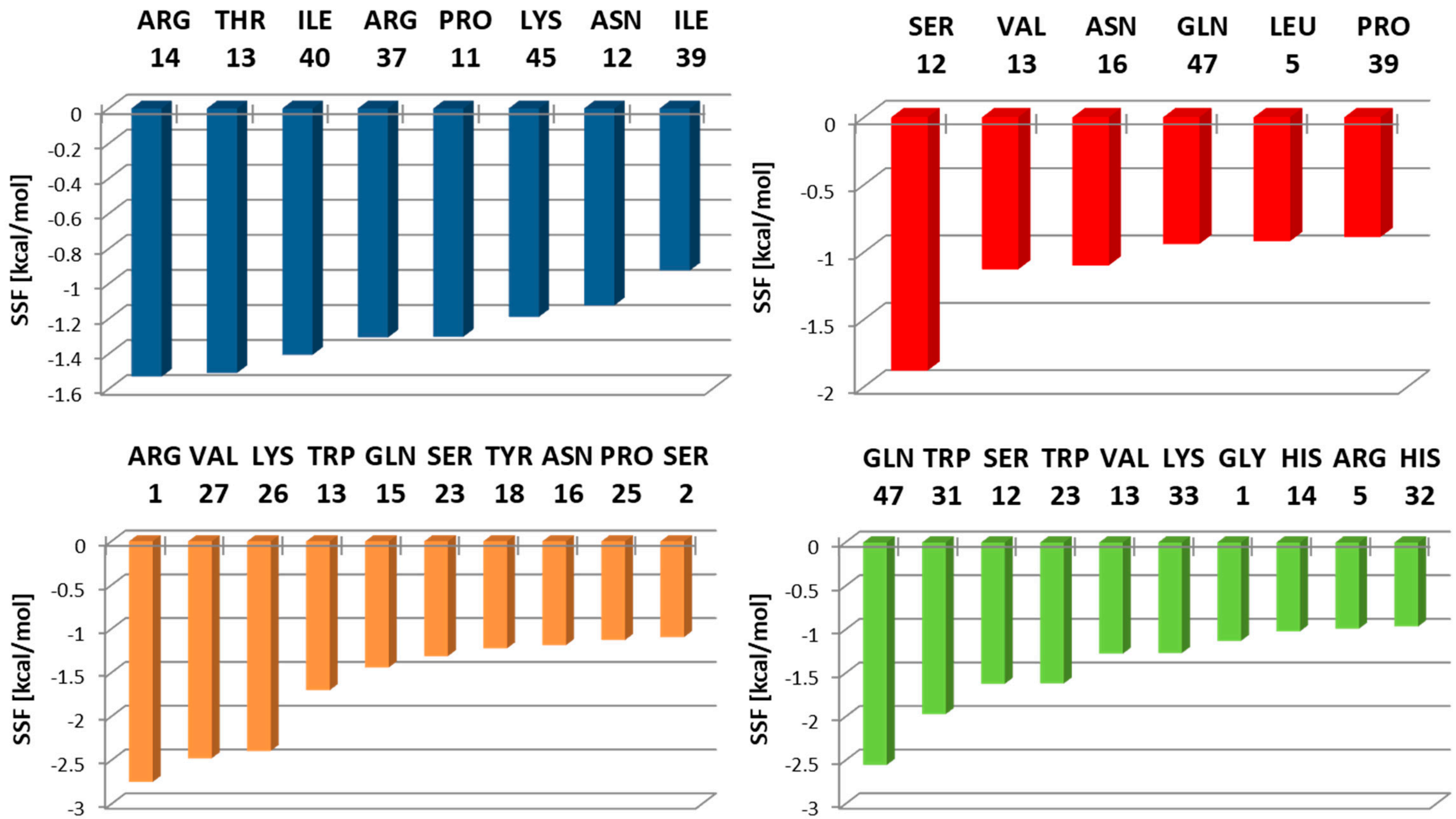

Figure S2. Contributions of toxins residues to the binding energy to NavPaS. Binding energy was calculated with Smina Scoring function (SSF) and is shown in kcal/mol. Amino acid residues of Av1 are shown in blue, Av2 – red, Av3 – orange and CgNa – green.

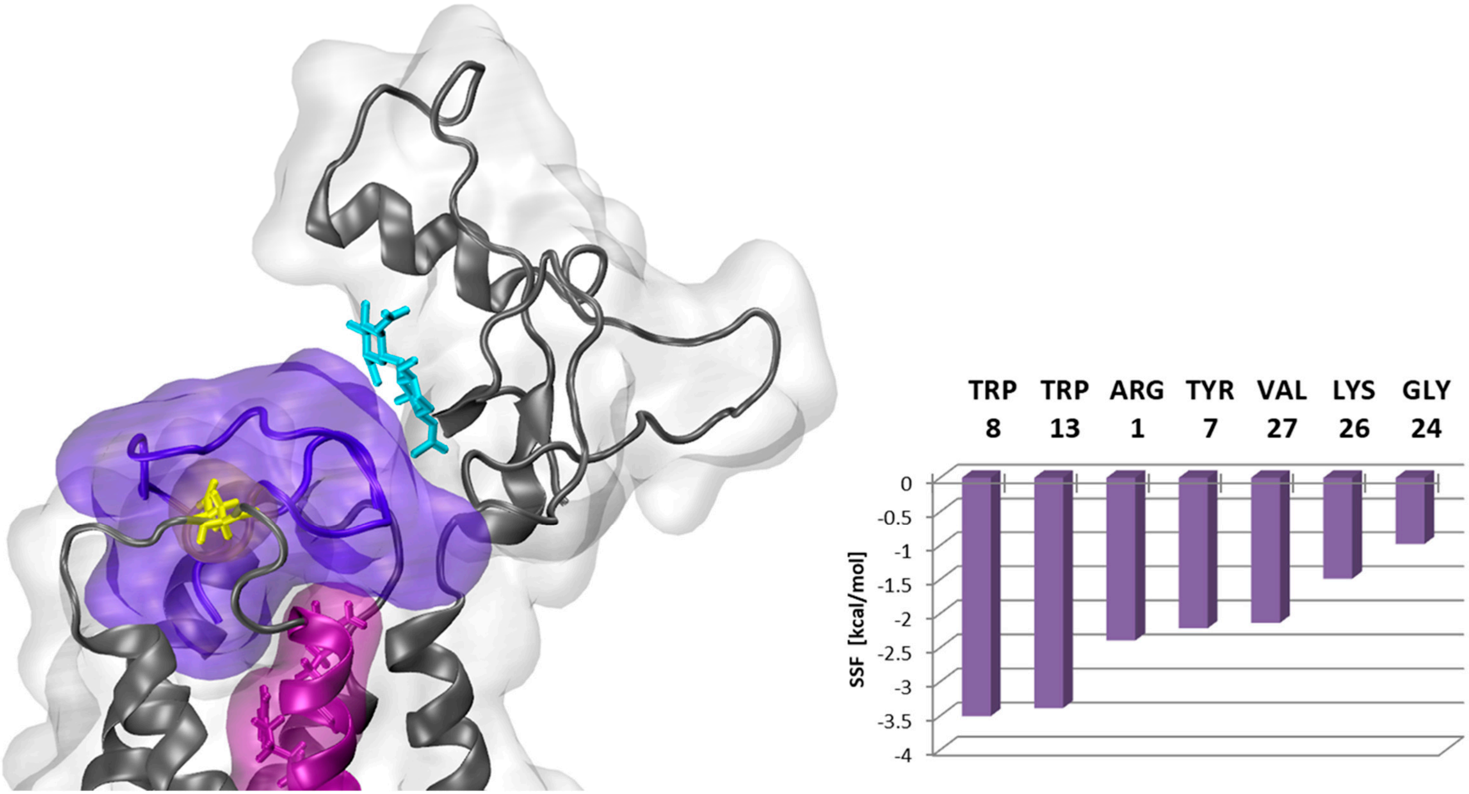

Figure S3. Binding mode of Av3 alternative pose – Av3' in the NavPaS site 3 region. Sodium channel is shown in grey (cartoon and surface representations) with voltage sensing helix S4 in purple, Glu1255 is in yellow and NAG (N-acetylglucosamine or 2-acetamino-2-deoxy-beta-D-glucopyranose) in cyan licorice representation. Av3 toxin is shown in violet cartoon and surface representation. In the right panel contributions of toxins residues to the binding energy (SSF, in kcal/mol) are shown.

Table S1a. Residue-Residue Contact Score (RRCS) values for possible toxin-NavPaS channel (amino acid residues) contacts.

**Av1**	**NavPas**	**RRCS**	**Av2**	**NavPas**	**RRCS**	**Av3**	**NavPas**	**RRCS**	**Av3'**	**NavPas**	**RRCS**	**CgNa**	**NavPas**	**RRCS**
		Σ=122.09			Σ=85.99			Σ=152.81			Σ=145.91			Σ=119.13
40 ILE	303 ASP	14.45	14 ARG	1193 GLY	10.30	1 ARG	331 SER	11.42	8 TRP	1259 ILE	9.65	47 GLN	345 GLN	19.43
9 ASP	1255 GLU	9.94	35 LYS	1255 GLU	9.69	13 TRP	1257 TYR	9.46	7 TYR	1248 LEU	7.87	8 SER	1261 PRO	8.00
40 ILE	306 TRP	7.72	16 ASN	345 GLN	6.56	1 ARG	330 ASN	8.74	1 ARG	1194 GLN	7.78	5 ARG	1255 GLU	7.76
12 ASN	1203 ASP	7.20	22 ILE	1256 LYS	5.45	16 ASN	345 GLN	8.22	26 LYS	1203 ASP	7.72	33 LYS	1196 MET	6.77
13 THR	1199 SER	5.24	47 GLN	301 PHE	4.88	27 VAL	1190 ASP	7.61	24 GLY	1200 GLU	6.87	9 ASP	1255 GLU	6.73
13 THR	1194 GLN	5.18	5 LEU	1255 GLU	4.70	1 ARG	388 ALA	7.00	9 GLY	1255 GLU	6.31	6 CYS	1255 GLU	6.48
8 SER	1261 PRO	5.03	14 ARG	1192 TYR	4.45	2 SER	1262 THR	5.82	1 ARG	345 GLN	6.31	33 LYS	1199 SER	6.38
14 ARG	1194 GLN	4.24	40 THR	1252 ASP	4.33	1 ARG	392 HID	5.22	27 VAL	1203 ASP	5.89	5 ARG	1256 LYS	6.19
45 LYS	1255 GLU	4.05	16 ASN	281 MET	3.12	23 SER	1199 SER	5.12	10 GLY	1255 GLU	5.18	7 ASP	1255 GLU	5.43
45 LYS	1253 VAL	3.88	10 GLY	1261 PRO	3.10	26 LYS	1190 ASP	4.66	19 PRO	1255 GLU	5.09	31 TRP	347 TYR	4.06
6 CYS	1255 GLU	3.81	12 SER	278 GLN	2.97	1 ARG	387 SER	4.02	13 TRP	283 VAL	4.79	1 GLY	303 ASP	3.93
12 ASN	1199 SER	3.31	14 ARG	1194 GLN	2.88	23 SER	1203 ASP	4.00	23 SER	1200 GLU	4.06	32 HIS	1194 GLN	3.65
7 LYS	1256 LYS	3.16	12 SER	281 MET	2.54	27 VAL	1265 ARG	3.88	1 ARG	1193 GLY	4.02	12 SER	283 VAL	2.71
14 ARG	1193 GLY	3.08	17 THR	345 GLN	1.99	26 LYS	1203 ASP	3.84	7 TYR	1247 GLY	3.66	47 GLN	1192 TYR	2.53
14 ARG	281 MET	3.07	12 SER	1194 GLN	1.80	1 ARG	329 GLY	3.81	7 TYR	1255 GLU	3.61	33 LYS	1200 GLU	2.52
25 PHE	301 PHE	2.77	15 GLY	1192 TYR	1.72	15 GLN	286 GLN	3.76	8 TRP	1261 PRO	3.58	12 SER	345 GLN	2.02
1 GLY	1200 GLU	2.74	10 GLY	1265 ARG	1.72	18 TYR	1199 SER	3.74	13 TRP	279 ILE	3.13	14 HIS	392 HIS	1.83
14 ARG	1192 TYR	2.70	12 SER	1190 ASP	1.71	26 LYS	1202 LEU	3.42	23 SER	1196 MET	2.97	38 TYR	1196 MET	1.75
9 ASP	1261 PRO	2.69	12 SER	1265 ARG	1.54	15 GLN	283 VAL	3.35	13 TRP	388 ALA	2.86	23 TRP	307 PHE	1.71
1 GLY	1196 MET	1.84	47 GLN	303 ASP	1.48	5 PRO	1255 GLU	3.14	24 GLY	1199 SER	2.85	13 VAL	388 ALA	1.70
11 PRO	1264 LEU	1.80	39 PRO	1254 ILE	1.40	23 SER	1200 GLU	3.02	8 TRP	1258 PHE	2.73	16 ASN	1257 TYR	1.67
39 ILE	306 TRP	1.74	38 GLY	1253 VAL	1.40	24 GLY	1203 ASP	2.87	1 ARG	1192 TYR	2.64	33 LYS	1203 ASP	1.59
15 GLY	1265 ARG	1.68	11 PRO	1265 ARG	1.16	22 CYS	1196 MET	2.85	6 CYS	1255 GLU	2.63	12 SER	286 GLN	1.51
37 ARG	345 GLN	1.64	13 VAL	1194 GLN	0.80	2 SER	1261 PRO	2.46	8 TRP	1255 GLU	2.55	31 TRP	307 PHE	1.38
10 GLY	1261 PRO	1.64	16 ASN	1192 TYR	0.76	27 VAL	278 GLN	2.42	26 LYS	1200 GLU	2.33	13 VAL	331 SER	1.27
14 ARG	1191 HIS	1.60	38 GLY	1254 ILE	0.71	20 GLU	1196 MET	2.41	18 TYR	1255 GLU	2.28	30 GLY	1193 GLY	1.26

45 LYS	1252 ASP	1.47
45 LYS	1254 ILE	1.44
11 PRO	1265 ARG	1.36
2 ALA	1196 MET	1.25
8 SER	1255 GLU	1.20
9 ASP	1253 VAL	1.14
18 MET	1196 MET	1.04
41 GLY	303 ASP	0.89
40 ILE	301 PHE	0.88
19 SER	1255 GLU	0.77
7 LYS	1255 GLU	0.73
34 CYS	1255 GLU	0.73
32 ASN	1254 ILE	0.66
11 PRO	1261 PRO	0.61
11 PRO	1203 ASP	0.55
15 GLY	281 MET	0.44
32 ASN	1256 LYS	0.39
33 ASN	1256 LYS	0.21
13 THR	1203 ASP	0.09
46 GLN	1252 ASP	0.06

4 CYS	1256 LYS	0.69
39 PRO	1256 LYS	0.64
38 GLY	1255 GLU	0.49
30 GLY	301 PHE	0.26
6 CYS	1255 GLU	0.21
3 PRO	1256 LYS	0.18
44 CYS	1256 LYS	0.17
13 VAL	281 MET	0.10
13 VAL	1192 TYR	0.09
38 GLY	1252 ASP	0.01

3 CYS	1261 PRO	2.32
1 ARG	286 GLN	2.27
26 LYS	1268 ARG	2.26
15 GLN	345 GLN	2.04
27 VAL	1194 GLN	1.75
21 GLY	1196 MET	1.72
18 TYR	1194 GLN	1.68
25 PRO	1265 ARG	1.51
8 TRP	1255 GLU	1.38
26 LYS	1206 ASN	1.28
3 CYS	1265 ARG	1.19
1 ARG	1262 THR	0.85
27 VAL	281 MET	0.82
15 GLN	387 SER	0.78
3 CYS	27 VAL	0.67
25 PRO	1261 PRO	0.57
26 LYS	1265 ARG	0.55
21 GLY	1199 SER	0.53
1 ARG	334 ALA	0.47
15 GLN	388 ALA	0.34
22 CYS	1200 GLU	0.28
3 CYS	1262 THR	0.26
1 ARG	389 GLY	0.25
26 LYS	1199 SER	0.23
17 CYS	281 MET	0.20
2 SER	1260 SER	0.18
3 CYS	278 GLN	0.12
26 LYS	1194 GLN	0.05

27 VAL	1206 ASN	2.07
26 LYS	1204 TYR	1.93
7 TYR	1253 VAL	1.83
25 PRO	1199 SER	1.82
11 CYS	1261 PRO	1.81
24 GLY	1203 ASP	1.77
27 VAL	1248 LEU	1.76
13 TRP	286 GLN	1.66
8 TRP	1247 GLY	1.57
1 ARG	1191 HIS	1.53
25 PRO	1203 ASP	1.48
24 GLY	1196 MET	1.19
13 TRP	387 SER	0.82
13 TRP	345 GLN	0.80
20 GLU	1251 SER	0.76
8 TRP	1253 VAL	0.73
1 ARG	281 MET	0.49
18 TYR	1196 MET	0.48
27 VAL	1264 LEU	0.46
14 GLY	345 GLN	0.42
12 PRO	1261 PRO	0.42
12 PRO	1262 THR	0.16
5 PRO	1255 GLU	0.15
25 PRO	1196 MET	0.13
13 TRP	392 HIS	0.12
26 LYS	1199 SER	0.12
8 TRP	1264 LEU	0.10

11 PRO	1265 ARG	1.25
23 TRP	306 TRP	1.15
31 TRP	1194 GLN	0.71
23 TRP	303 ASP	0.69
11 PRO	1262 THR	0.65
5 ARG	1254 ILE	0.49
12 SER	279 ILE	0.48
46 LYS	345 GLN	0.48
24 VAL	303 ASP	0.48
31 TRP	345 GLN	0.46
32 HIS	1193 GLY	0.35
13 VAL	387 SER	0.33
36 ASP	1200 GLU	0.31
16 ASN	1256 LYS	0.24
16 ASN	1255 GLU	0.21
11 PRO	1261 PRO	0.20
7 ASP	1253 VAL	0.17
38 TYR	1200 GLU	0.07
13 VAL	1262 THR	0.04
2 VAL	303 ASP	0.04
14 HIS	331 SER	0.03
12 SER	388 ALA	0.02
47 GLN	344 LEU	0.02
32 HIS	1195 SER	0.01

Table S1b. Residue-Residue Contact Score (RRCS) values for possible toxin-NavPaS channel NAG contacts.

Av1	NAG	RRCS	Av2	NAG	RRCS	Av3	NAG	RRCS	Av3'	NAG	RRCS	CgNa	NAG	RRCS
		Σ=26.63			Σ=19.93			Σ=13.92			Σ=2.53			Σ=38.08
39 ILE	1602 NAG	7.40	19 SER	1602 NAG	8.80	10 GLY	1601 NAG	7.07	14 GLY	1601 NAG	1.48	19 SER	1601 NAG	7.44
37 ARG	1601 NAG	5.15	21 ILE	1602 NAG	3.85	15 GLN	1601 NAG	2.50	2 SER	1602 NAG	0.19	3 HYP	1602 NAG	5.53
36 GLY	1602 NAG	4.57	19 SER	1601 NAG	2.34	16 ASN	1602 NAG	1.87	13 TRP	1601 NAG	0.09	21 THR	1602 NAG	4.49
40 ILE	1602 NAG	3.57	17 THR	1601 NAG	2.27	16 ASN	1601 NAG	1.04	15 GLN	1601 NAG	0.14	47 GLN	1602 NAG	3.95
37 ARG	1602 NAG	2.43	1 GLY	1601 NAG	1.32	14 GLY	1601 NAG	0.45	15 GLN	1602 NAG	0.00	1 GLY	1602 NAG	3.79
41 GLY	1602 NAG	2.25	21 ILE	1601 NAG	0.46	9 GLY	1601 NAG	0.38	12 PRO	1601 NAG	0.62	19 SER	1602 NAG	3.77
35 GLU	1601 NAG	0.56	18 LEU	1601 NAG	0.34	1 ARG	1601 NAG	0.35				47 GLN	1601 NAG	2.09
38 ALA	1602 NAG	0.42	47 GLN	1602 NAG	0.22	10 GLY	1602 NAG	0.17				18 LEU	1602 NAG	1.72
36 GLY	1601 NAG	0.28	3 PRO	1601 NAG	0.19	11 CYS	1601 NAG	0.09				18 LEU	1602 NAG	1.72
42 TYR	1602 NAG	0.01	1 GLY	1602 NAG	0.15							20 GLY	1602 NAG	1.42
												2 VAL	1602 NAG	1.21
												18 LEU	1601 NAG	0.61
												17 THR	1601 NAG	0.20
												13 VAL	1601 NAG	0.13

DI/SS2-S6 fragment

$PaNa_V1$ ALRSAGPWHILFF[396]

$DmNa_V1$ VLRAAGPWHMLFF[409]

$TcNa_V1$ VLRAAGPWHMLFF[408]

$DpNa_V1$ VLRTAGPWHMLFF[325]

Figure S4. Multiple sequence alignment of DI/SS2-S6 region of selected arthropod voltage-gated sodium (Nav) channels. Species abbreviations: Insects: Pa - *Periplaneta americana* (cockroach), Dm - *Drosophila melanogaster* (fruitfly), Tc - *Tribolium castaneum* (red flour beetle), Crustacean: Dp - *Daphnia pulex* (water flea,). The highly conserved histidine is marked in red.

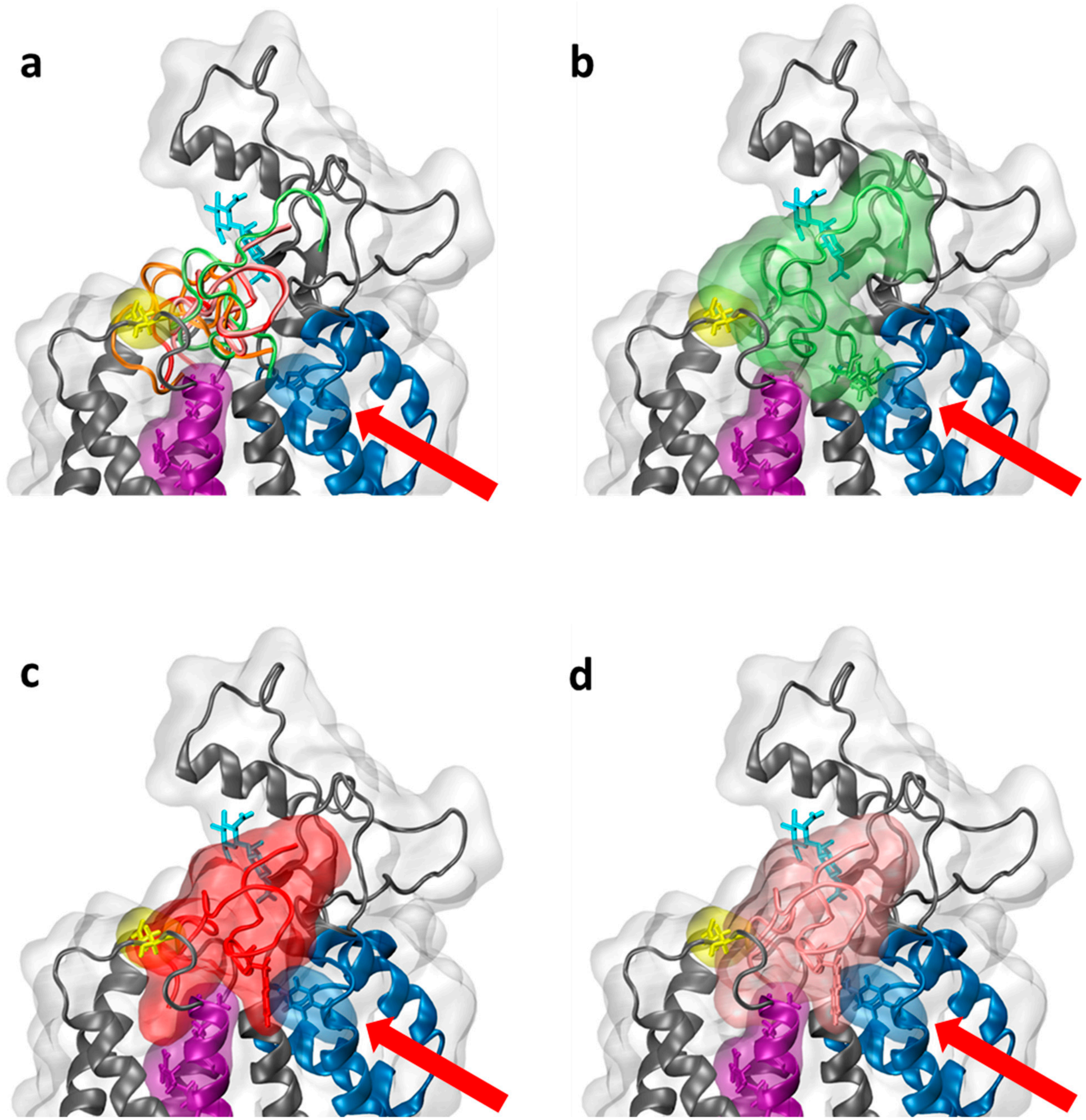
a
b
c
d

Figure S5. Representations of Av3 toxin binding modes to the site 3 of NavPaS channel H392X mutant variants. (a) the lowest energy poses of Av3 binding to NavPaS WT (orange) and H392 mutants. (b) Av3 (green) docked to the H392A (c) Av3 (red) docked to H392F (d) Av3 (pink) docked to H392Y. In purple helix S4 is presented, E1255 is shown in a yellow licorice and surface representation and NAG (N-acetylglucosamine) is depicted in cyan licorice. Pore forming region is in blue with H392X residues marked in licorice and surface representation and its position indicated by a red arrow.

Table S2. Parameters (*) characterizing interactions of Av3 anemone toxin with NavPaS channel wild type (WT) and H392X mutant variants.

**Parameter**	**Av3 WT**	**Av3′ WT**	**Av3 H392A**	**Av3 H392F**	**Av3 H392T**
SSF [kcal/mol]	**-9.72**	**-8.85**	**-8.86**	**-9.41**	**-9.05**
Total RRCS (AA)	152.80	145.90	151.31	145.81	157.49
Total RRCS for NAG	13.90	2.53	19.18	24.12	22.34
RRCS S4 only	21.84	6.51	36.08	8.86	16.61
Toxin AA in interface [%]	85	78	92.6	85.2	88.9
NAG atoms in interface [%]	50	46	64.25	67.85	67.85
ASA_TI [%]	46	45	47.3	53.0	52.7
ASA_NavI [%]	1.40	1.40	1.5	1.6	1.6
% BSA Glu1255	43	89	55	54	47
% BSA Arg1265 (S4)	94	31	60	70	92
% BSA Arg1268 (S4)	83	22	0	83	51
% BSA NAG	25	20	27	26	27
% BSA X392	50	16	94	74	70

(*) for definitions see main text

Table S3. Residue (# H392X NavPaS) - residue (#Av3 toxin) interactions responsible for anemone toxin binding to NavPaS mutant variants channel. Interaction types: pc - π-cation, sb – salt bridge, hbbb – hydrogen bond backbone-backbone, hbss – hydrogen bond side chain-side chain, hbsb – hydrogen bond side chain-backbone.

	**H392A NavPaS**	**Av3**		**H392F NavPaS**	**Av3**		**H392Y NavPaS**	**Av3**
			pc	TYR 1192	LYS 26			
						sb	ASP 1190	LYS 26
						sb	ASP 1190	LYS 26
hbbb	CYS 337	LYS 26	hbsb	SER 387	TYR 7	hbsb	GLY 335	ARG 1
hbss	SER 1260	ASN 16	hbsb	TYR 1257	ASN 16	hbss	ASP 1190	LYS 26
hbsb	ASN 330	VAL 27	hbss	GLN 286	TYR 7	hbsb	CYS 337	ARG 1
hbsb	ARG 1265	PRO 12	hbsb	CYS 337	ARG 1	hbsb	MET 281	TYR 7
hbsb	LYS 1256	PRO 19	hbsb	GLY 335	ARG 1	hbss	ASN 330	ARG 1
hbsb	ASN 330	TYR 18	hbsb	SER 1199	LYS 26	hbsb	GLN 345	SER 23
hbsb	THR 1262	ARG 1	hbsb	HIS 1191	LYS 26	hbsb	GLY 329	ARG 1
hbbb	ILE 279	ARG 1	hbsb	SER 1199	LYS 26	hbsb	CYS 337	ARG 1
hbsb	AL388	ARG 1	hbsb	CYS 337	ARG 1	hbsb	MET 281	TYR 7
hbss	GLN 286	SER 2	hbss	ASN 330	ARG 1	hbsb	THR 1262	TYR 7
hbsb	ARG 1265	PRO 12	hbsb	GLY 329	ARG 1	hbsb	GLN 345	SER 23
			hbsb	THR 1262	TYR 7	hbsb	GLY 329	ARG 1
hbls	TYR 7	NAG 1602	hbsb	GLN 1194	PRO 25	hbss	ARG 1265	VAL 27
hbls	TYR 7	NAG 1601	hbsb	GLU 1255	ASN 16	hbss	ASP 1190	LYS 26
hbls	TYR 7	NAG 1602	hbsb	GLY 329	ARG 1			
						hbls	TYR 18	NAG 1602
			hblb	ARG 1	NAG 1601	hbls	TYR 18	NAG 1601
			hblb	SER 2	NAG 1601	hblb	SER 2	NAG 1601
			hbls	TYR 18	NAG 1602	hblb	ARG 1	NAG 1601
			hbls	TYR 18	NAG 1601	hbls	TYR 18	NAG 1602
			hbls	TYR 18	NAG 1602			

Table S4a. Residue-Residue Contact Score values for possible Av3 toxin-NavPaS channel H392X variants (amino acid residues) contacts.

**Av3**	**NavPaS H392A**	**RRCS**	**Av3**	**NavPaS H392F**	**RRCS**	**Av3**	**NavPaS H392T**	**RRCS**
		Σ=151.31			Σ=145.81			Σ=157.49
15 GLN	1262 THR	8.07	27 VAL	1190 ASP	7.58	23 SER	345 GLN	11.39
27 VAL	330 ASN	8.00	1 ARG	337 CYS	5.98	1 ARG	337 CYS	7.75
1 ARG	279 ILE	7.95	19 PRO	1255 GLU	5.54	16 ASN	1257 TYR	7.73
16 ASN	1260 SER	7.31	16 ASN	1257 TYR	5.28	27 VAL	1265 ARG	7.54
20 GLU	1256 LYS	7.19	25 PRO	1199 SER	4.71	19 PRO	1255 GLU	7.38
18 TYR	330 ASN	6.85	16 ASN	1255 GLU	4.57	16 ASN	1256 LYS	7.27
1 ARG	392 ALA	6.56	1 ARG	330 ASN	4.55	7 TYR	281 MET	5.92
16 ASN	1257 TYR	5.74	7 TYR	388 ALA	4.42	7 TYR	279 ILE	5.67
1 ARG	388 ALA	5.54	25 PRO	1194 GLN	4.29	27 VAL	281 MET	5.07
8 TRP	1255 GLU	5.02	26 LYS	1194 GLN	4.22	1 ARG	330 ASN	4.74
27 VAL	336 MET	4.97	25 PRO	1196 MET	4.22	1 ARG	329 GLY	4.65
19 PRO	1256 LYS	4.72	26 LYS	1199 SER	4.04	27 VAL	278 GLN	4.51
25 PRO	339 PRO	4.63	1 ARG	336 MET	3.95	5 PRO	331 SER	3.79
27 VAL	329 GLY	3.96	27 VAL	1202 LEU	3.84	12 PRO	332 SER	3.69
13 TRP	1192 TYR	3.56	1 ARG	329 GLY	3.74	7 TYR	1262 THR	3.59
19 PRO	1257 TYR	3.54	13 TRP	330 ASN	3.62	13 TRP	332 SER	3.57
26 LYS	337 CYS	3.49	7 TYR	283 VAL	3.59	26 LYS	1199 SER	3.54
26 LYS	336 MET	3.42	7 TYR	286 GLN	3.42	5 PRO	330 ASN	3.17
12 PRO	1265 ARG	3.37	13 TRP	332 SER	3.38	23 SER	1192 TYR	3.12
1 ARG	280 TYR	3.22	7 TYR	1262 THR	3.24	8 TRP	392 TYR	2.91
15 GLN	1260 SER	3.21	5 PRO	331 SER	3.04	26 LYS	1190 ASP	2.89
16 ASN	1259 ILE	3.06	12 PRO	332 SER	2.94	1 ARG	343 CYS	2.88
13 TRP	281 MET	2.66	8 TRP	392 PHE	2.87	25 PRO	1192 TYR	2.75
25 PRO	341 TYR	2.50	27 VAL	1199 SER	2.83	13 TRP	330 ASN	2.52

2 SER	388 ALA	2.44	13 TRP	331 SER	2.82	13 TRP	331 SER	2.42
14 GLY	1262 THR	2.44	27 VAL	1203 ASP	2.65	1 ARG	336 MET	2.30
2 SER	283 VAL	2.42	16 ASN	1256 LYS	2.41	7 TYR	278 GLN	2.20
11 CYS	1261 PRO	2.27	19 PRO	1261 PRO	2.40	20 GLU	1261 PRO	2.09
1 ARG	391 TRP	2.09	7 TYR	387 SER	2.23	25 PRO	1194 GLN	2.07
14 GLY	1261 PRO	2.00	23 SER	1192 TYR	2.08	19 PRO	1261 PRO	1.92
3 CYS	331 SER	1.75	26 LYS	281 MET	2.01	1 ARG	335 GLY	1.85
1 ARG	1262 THR	1.64	5 PRO	330 ASN	2.01	3 CYS	330 ASN	1.85
16 ASN	1261 PRO	1.60	25 PRO	1195 SER	1.98	24 GLY	1192 TYR	1.83
2 SER	286 GLN	1.53	3 CYS	330 ASN	1.94	7 TYR	283 VAL	1.78
27 VAL	331 SER	1.34	7 TYR	392 PHE	1.90	26 LYS	1194 GLN	1.65
15 GLN	1261 PRO	1.34	8 TRP	1262 THR	1.85	7 TYR	1265 ARG	1.63
10 GLY	1255 GLU	1.33	1 ARG	335 GLY	1.83	27 VAL	1190 ASP	1.54
12 PRO	1261 PRO	1.20	1 ARG	343 CYS	1.80	26 LYS	1203 ASP	1.35
1 ARG	389 GLY	0.92	26 LYS	1191 HIS	1.63	7 TYR	392 TYR	1.15
2 SER	331 SER	0.89	27 VAL	278 GLN	1.62	4 CYS	330 ASN	1.12
27 VAL	334 ALA	0.76	26 LYS	1192 TYR	1.39	8 TRP	1262 THR	1.11
27 VAL	335 GLY	0.74	27 VAL	1194 GLN	1.37	1 ARG	341 TYR	1.08
25 PRO	338 PRO	0.73	27 VAL	1265 ARG	1.30	16 ASN	1255 GLU	0.88
25 PRO	291 HIS	0.65	20 GLU	1255 GLU	1.18	10 GLY	1257 TYR	0.84
2 SER	279 ILE	0.65	26 LYS	1203 ASP	1.03	25 PRO	1191 HIS	0.84
25 PRO	337 CYS	0.51	1 ARG	341 TYR	0.97	7 TYR	280 TYR	0.82
13 TRP	345 GLN	0.48	10 GLY	1257 TYR	0.92	1 ARG	328 CYS	0.77
14 GLY	1265 ARG	0.30	26 LYS	1193 GLY	0.88	25 PRO	281 MET	0.75
23 SER	330 ASN	0.13	4 CYS	330 ASN	0.88	1 ARG	342 THR	0.67
26 LYS	339 PRO	0.13	11 CYS	331 SER	0.71	11 CYS	331 SER	0.53
2 SER	345 GLN	0.13	15 GLN	1256 LYS	0.59	15 GLN	1256 LYS	0.53
3 CYS	388 ALA	0.12	2 SER	330 ASN	0.56	11 CYS	330 ASN	0.38
9 GLY	1255 GLU	0.10	1 ARG	342 THR	0.41	20 GLU	1265 ARG	0.35
17 CYS	331 SER	0.07	11 CYS	330 ASN	0.32	2 SER	330 ASN	0.33
16 ASN	1255 GLU	0.04	13 TRP	329 GLY	0.20	26 LYS	1265 ARG	0.30

5 PRO	330 ASN	0.03	6 CYS	1261 PRO	0.07	13 TRP	329 GLY	0.17
10 GLY	1261 PRO	0.02				27 VAL	1194 GLN	0.14
						11 CYS	332 SER	0.11
						8 TRP	331 SER	0.09

Table S4b. Residue-Residue Contact Score values for possible toxin-NavPaS channel NAG contacts.

**Av3**	**NAG**	**RRCS**	**Av3**	**NAG**	**RRCS**	**Av3**	**NAG**	**RRCS**
		Σ=19.18			Σ=24.12			Σ=22.34
7 TYR	1601 NAG	6.09	2 SER	1601 NAG	8.48	2 SER	1601 NAG	7.40
7 TYR	1602 NAG	5.45	18 TYR	1601 NAG	7.48	18 TYR	1601 NAG	7.38
5 PRO	1601 NAG	5.29	1 ARG	1601 NAG	2.60	1 ARG	1601 NAG	2.32
23 SER	1601 NAG	1.62	18 TYR	1602 NAG	2.39	18 TYR	1602 NAG	2.32
4 CYS	1601 NAG	0.26	3 CYS	1601 NAG	1.40	3 CYS	1601 NAG	1.33
3 CYS	1601 NAG	0.23	5 PRO	1601 NAG	1.33	5 PRO	1601 NAG	1.15
6 CYS	1601 NAG	0.12	6 CYS	1601 NAG	0.44	6 CYS	1601 NAG	0.44
24 GLY	1601 NAG	0.12						

Table S5. Values of SMINA Scoring Function [kcal/mol] for anemones toxins binding to NavPaS channel variants (the lowest energy poses of 5 repetitions up to 100 poses).

**Channel variant**	**Av1**	**Av2**	**Av3**	**CgNa**
**WT**	**-4.89**	**-4.98**	**-9.72**	**-5.53**
**D1252E**	-	-4.81	-8.88	-5.48
**D1252R**	-	-4.66	-8.95	-5.81
**D1252A**	-	-4.90	-9.30	-5.49
